# Marek’s disease virus U_S_3 protein kinase phosphorylates chicken HDAC 1 and 2 and regulates viral replication and pathogenesis

**DOI:** 10.1371/journal.ppat.1009307

**Published:** 2021-02-17

**Authors:** Yifei Liao, Blanca Lupiani, Mohammad AI-Mahmood, Sanjay M. Reddy

**Affiliations:** Department of Veterinary Pathobiology, College of Veterinary Medicine & Biomedical Sciences, Texas A&M University, College Station, Texas, United States of America; Emory Vaccine Center, UNITED STATES

## Abstract

Marek’s disease virus (MDV) is a potent oncogenic alphaherpesvirus that elicits a rapid onset of malignant T-cell lymphomas in chickens. Three MDV types, including GaHV-2 (MDV-1), GaHV-3 (MDV-2) and MeHV-1 (HVT), have been identified and all encode a U_S_3 protein kinase. MDV-1 U_S_3 is important for efficient virus growth *in vitro*. To study the role of U_S_3 in MDV replication and pathogenicity, we generated an MDV-1 U_S_3-null virus and chimeric viruses by replacing MDV-1 U_S_3 with MDV-2 or HVT U_S_3. Using MD as a natural virus-host model, we showed that both MDV-2 and HVT U_S_3 partially rescued the growth deficiency of MDV-1 U_S_3-null virus. In addition, deletion of MDV-1 U_S_3 attenuated the virus resulting in higher survival rate and lower MDV specific tumor incidence, which could be partially compensated by MDV-2 and HVT U_S_3. We also identified chicken histone deacetylase 1 (chHDAC1) as a common U_S_3 substrate for all three MDV types while only U_S_3 of MDV-1 and MDV-2 phosphorylate chHDAC2. We further determined that U_S_3 of MDV-1 and HVT phosphorylate chHDAC1 at serine 406 (S406), while MDV-2 U_S_3 phosphorylates S406, S410, and S415. In addition, MDV-1 U_S_3 phosphorylates chHDAC2 at S407, while MDV-2 U_S_3 targets S407 and S411. Furthermore, biochemical studies show that MDV U_S_3 mediated phosphorylation of chHDAC1 and 2 affect their stability, transcriptional regulation activity, and interaction network. Using a class I HDAC specific inhibitor, we showed that MDV U_S_3 mediated phosphorylation of chHDAC1 and 2 is involved in regulation of virus replication. Overall, we identified novel substrates for MDV U_S_3 and characterized the role of MDV U_S_3 in MDV pathogenesis.

## Author summary

Marek’s disease virus (MDV) is a highly contagious and oncogenic avian alphaherpesvirus that causes T-cell lymphomas in chickens. Alphaherpesviruses encoded U_S_3 is a multifunctional protein kinase involved in viral replication, apoptosis resistance, and cell-to-cell spread. In this study, we evaluated the importance of MDV U_S_3 in regulating MDV replication and pathogenesis in chickens. Our results provide first evidence that MDV U_S_3 protein kinase is involved in the replication and pathogenicity of MDV in its natural host. We also identified chicken histone deacetylase 1 and 2 (chHDAC1 and 2) as novel substrates of U_S_3 for MDV and characterized the potential impacts of MDV U_S_3 induced phosphorylation in their protein stability, transcriptional regulation and protein interactions; to our knowledge, this is the first comparative study of the functions of U_S_3 from all three MDV types. This is an important finding towards a better understanding of the functions of alphaherpesviruses encoded U_S_3 protein kinase.

## Introduction

Marek’s disease virus (MDV), an avian alphaherpesvirus, is the etiological agent of Marek’s disease (MD) which is associated with rapid induction of T-cell lymphomas in chickens. Currently, three antigenically related MDVs have been identified and sequenced, including MDV-1 (also known as *Gallid alphaherpesvirus* 2 [GaHV-2]), MDV-2 (also known as *Gallid alphaherpesvirus* 3 [GaHV-3]), and turkey herpesvirus (HVT; also known as *Meleagrid alphaherpesvirus* type 1 [MeHV-1]) [1]. Only MDV-1 can cause tumors in infected chickens, while MDV-2 and HVT are naturally non-oncogenic viruses from chickens and turkeys, respectively. Attenuated MDV-1 along with MDV-2 and HVT have been used, alone or in combination, as vaccines to protect susceptible chickens from MD. MDV is classified as a member of the *Alphaherpesvirinae* subfamily, which also includes animal herpesviruses such as pseudorabies virus (PRV) and bovine herpesvirus type 1 (BHV-1), as well as human herpesviruses such as herpes simplex virus type 1 and 2 (HSV-1 and -2) and varicella zoster virus (VZV). The genome of MDV-1 is approximately 177 kb in length and encodes more than 100 genes [2]. Most MDV-1 genes have homologues in other alphaherpesviruses and share similar functions, but MDV encodes some unique genes such as *meq* and *vTR* which are directly involved in MDV oncogenicity [3,4].

Like other alphaherpesviruses, MDV encodes a U_S_3 serine/threonine protein kinase which has been shown to be involved in apoptosis resistance, actin stress fiber breakdown and cell-to-cell spread [5,6]. U_S_3 orthologs contain a conserved ATP-binding domain and a catalytic active domain, although the complete U_S_3 protein sequence similarity is variable [7]. It has been reported that U_S_3 is important for virus growth *in vitro*, as deletion of U_S_3 results in growth deficiency of HSV-1, PRV, VZV, and MDV [7]. In addition, various functions have been attributed to U_S_3, such as protein phosphorylation, apoptosis inhibition, virion morphogenesis, transcriptional regulation, and cytoskeletal re-arrangements [7,8]. Schumacher *et al*. demonstrated that MDV-1 U_S_3 interacts with and phosphorylates viral protein pp38, an MDV protein involved in early lytic infection in lymphocytes [6]. We have recently identified MDV-1 Meq and cellular cAMP response element-binding protein (CREB) as targets of MDV-1 U_S_3 [9]. In addition, we have shown that MDV-1 U_S_3 induced CREB phosphorylation up-regulates the transcription of *c-Fos* and several viral genes [9].

Histone deacetylases (HDACs) are a class of enzyme that can reverse the acetylation of histone lysine residues [10]. Currently, eighteen HDACs have been identified in mammals and are grouped into four classes: class I consists of HDAC1, 2, 3, and 8; class II consists of HDAC4, 5, 6, 7, 9, and 10; class III consists of seven NAD^+^-dependent Sirtuin 1–7; and class IV has only one member, HDAC11 [11]. Class I HDACs are expressed in all tissues and play crucial roles in tissue development, cell differentiation and proliferation, and cancer formation [11,12]. More specifically, HDAC1 and HDAC2 (HDAC1 and 2) are highly homologous proteins and show conserved or specific functions depending on the stimuli [11]. HDAC1 and 2 not only act as protein modifiers, but are in turn modified by other cellular regulators mainly through three mechanisms: post-translational modifications, protein interaction network, and subcellular localization [11]. Casein kinase II (CKII), the main upstream protein kinase of HDAC1 and 2, phosphorylates HDAC1 at serine 393 (S393), S421 and S423, and HDAC2 at S394, S422 and S424. CKII mediated phosphorylation of HDAC1 and 2 has been shown to regulate their catalytic activity and protein interactions [13–15]. HDAC1 and 2 are also phosphorylated by alphaherpesviruses U_S_3 serine/threonine protein kinase [16]. In addition, it has been reported that overexpression of HSV-1 U_S_3 affects the amount and distribution of HDAC1 [17]. The phosphorylation sites of HDAC1 and HDAC2 targeted by HSV-1 U_S_3 and VZV ORF66 (the U_S_3 ortholog in VZV) have been identified as S406 and S407, respectively [16,18]. It has been shown that inhibition of HDAC activity increases the plaque size and plaquing efficiency of U_S_3 null VZV and PRV, suggesting that U_S_3 targets HDACs to reduce viral genome silencing and allow for efficient viral replication [18].

In this study, using a natural virus-host model, we demonstrated that MDV-2 and HVT U_S_3 partially compensate the function of MDV-1 U_S_3 associated with MDV-1 replication and pathogenesis. We also investigated the role of MDV U_S_3 in the phosphorylation of chicken HDAC1 and 2 (chHDAC1 and 2). Our results show that U_S_3 of all three MDV types can phosphorylate chHDAC1: MDV-1 and HVT U_S_3 target chHDAC1 at S406, while MDV-2 U_S_3 has two additional target sites, S410 and S415. On the other hand, we found that MDV-1 U_S_3 phosphorylates chHDAC2 at S407 and MDV-2 U_S_3 phosphorylates chHDAC2 at S407 and S411, while chHDAC2 is not a substrate of HVT U_S_3. We further characterized the impact of MDV U_S_3 induced phosphorylation in regulating chHDAC1 and 2 functions. Our results show that MDV U_S_3 induced phosphorylation regulates protein stability, transcriptional regulation activity, and protein interactions of chHDAC1 and 2. We also found that U_S_3 from all three MDV types physically interacts with chHDAC1 and 2. Overall, our results reveal the role of U_S_3 from all three MDV types in regulating phosphorylation of chHDAC1 and 2, as well as in viral replication and pathogenesis.

## Results

### MDV-2 U_S_3 and HVT U_S_3 can partially rescue virus replication of MDV-1 U_S_3 null virus *in vitro*

**Fig 1:**
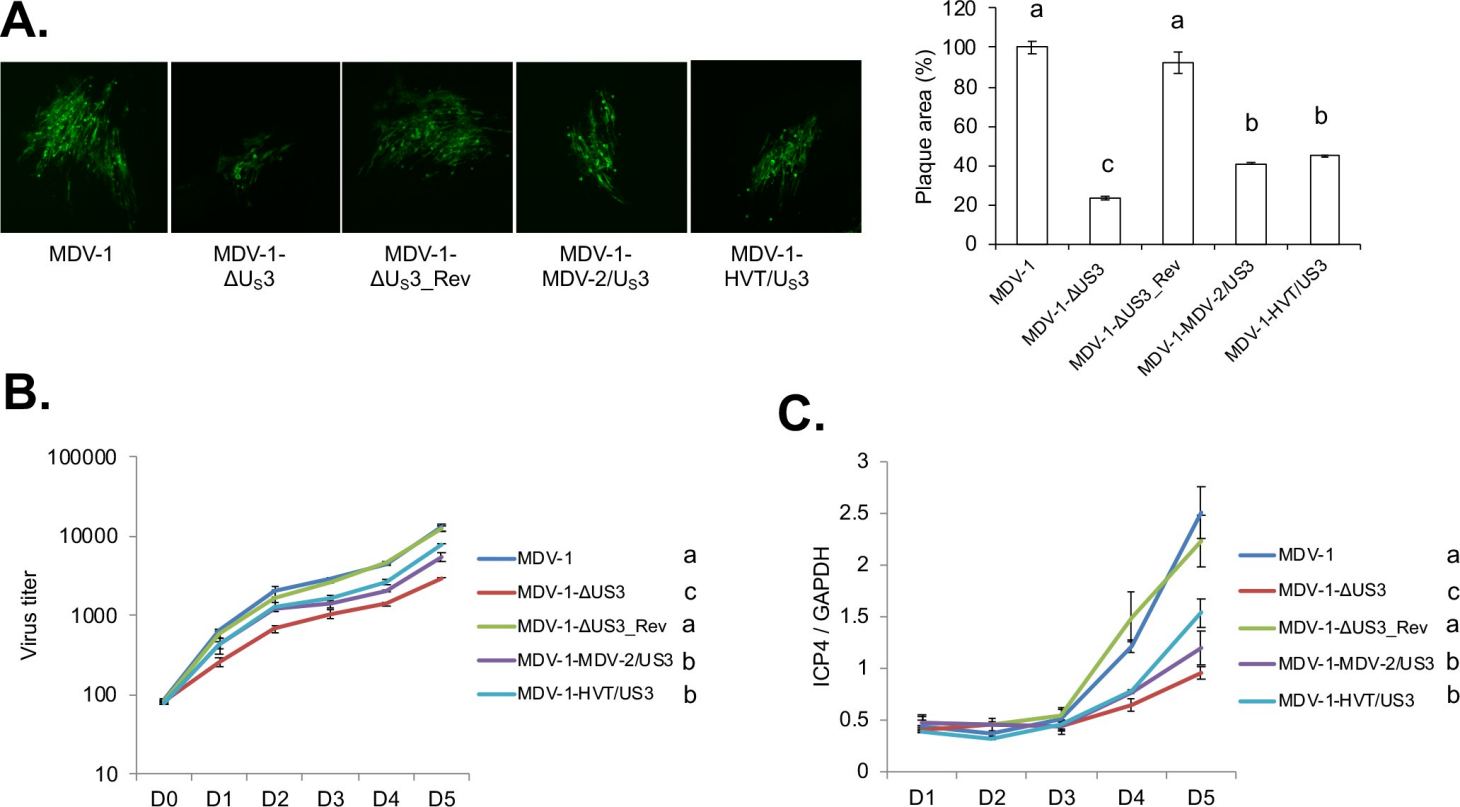
*In vitro* characterization of chimeric and revertant MDVs. (A) Chicken embryonic fibroblasts (CEF) were infected with parental MDV-1, MDV-1-ΔU_S_3, MDV-1-ΔU_S_3_Rev, MDV-1-MDV-2/U_S_3, or MDV-1-HVT/U_S_3 viruses and fixed at 7 days post infection. Immunofluorescence assay (IFA) was performed using MDV pp38 mouse monoclonal antibody and goat anti-mouse-Alexa Fluor 488 antibody. Plaques were visualized with a fluorescence microscope (left). For each virus, plaque sizes (50–100 plaques) were measured, and presented as mean plaque sizes relative to parental MDV-1 virus (right). The mean plaque size of parental MDV-1 virus was set to 100%. The error bar represents the standard deviation of two independent experiments. (B) CEF were infected with 100 plaque-forming units (PFU) of the indicated viruses. At the given days post infection, cells were trypsinized and serial 10-fold dilutions were co-seeded with fresh CEF. Numbers of plaques for each virus were counted, after IFA with pp38 antibody, and presented as mean virus titer. The error bar shows standard deviation of two independent experiments. (C) CEF were infected with 100 PFU of the indicated viruses. Cells were harvested daily until 5 days post infection and subjected to genomic DNA isolation. The genome copy number of each virus was determined by qPCR, using MDV *ICP4* and chicken *GAPDH* primers, and presented as the ratio of *ICP4* to *GAPDH* copy number with error bar representing the standard deviation. Significant difference between groups are marked as letters where different letters represent significant different at p<0.05.

It has been reported that HSV-2 U_S_3 could compensate some functions of HSV-1 U_S_3, such as apoptosis inhibition and cell morphology modulation, while also causing aberrant localization of UL34, a substrate of HSV-1 U_S_3 [19]. Here, we studied whether MDV-2 or HVT U_S_3 could rescue functions of MDV-1 U_S_3. For these studies we generated chimeric viruses by replacing MDV-1 U_S_3 with MDV-2 U_S_3 (MDV-1-MDV-2/U_S_3) or HVT U_S_3 (MDV-1-HVT/U_S_3), respectively. As previously reported [6], we observed that deletion of U_S_3 significantly reduced the plaque size of MDV-1 (Fig 1A). In addition, as expected, we found that MDV-2 U_S_3 and HVT U_S_3 could partially restore the plaque size of MDV-1-ΔU_S_3 virus, and the plaque size of MDV-1-MDV-2/U_S_3 and MDV-1-HVT/U_S_3 was not different (Fig 1A). *In vitro* growth kinetics of parental, U_S_3 deletion, chimeric and revertant viruses, show that U_S_3 deletion reduced the growth of MDV, as indicated by fewer plaque numbers, and that MDV-2 U_S_3 and HVT U_S_3 partially rescue the growth deficiency of U_S_3 deletion virus (Fig 1B). We speculate that reduced plaque numbers caused by U_S_3 deletion might be due to lower plaque forming efficiency or/and less viral genome copy numbers. To further distinguish these two possibilities, we analyzed the viral genome copy number after infecting CEF with chimeric and revertant viruses. Our results show that deletion of U_S_3 resulted in lower viral genome copy number, a phenotype which was partially restored by MDV-2 U_S_3 and HVT U_S_3 (Fig 1C). Based on these data we conclude that MDV-1 U_S_3 is involved in the replication of viral genome and MDV plaque forming efficiency.

### MDV-2 U_S_3 and HVT U_S_3 can partially rescue the replication and pathogenesis of MDV-1 U_S_3 null virus in chickens

To further characterizing the chimeric viruses *in vivo*, we examined the replication and pathogenesis of chimeric viruses in chickens a natural virus-host model. MDV establishes an early cytolytic infection 2–7 days post infection and switches to latent infection beginning 7–8 days post infection in lymphoid organs [20,21]. Within 2 weeks post infection, latently infected T lymphocytes become transformed and/or migrate to the skin where they infect feather follicle epithelium (FFE) to produce complete mature infectious virions [20]. At 3 to 4 weeks post infection, latent viruses reactivate resulting in a late cytolytic infection [20,21].

#### In vivo replication

**Fig 2:**
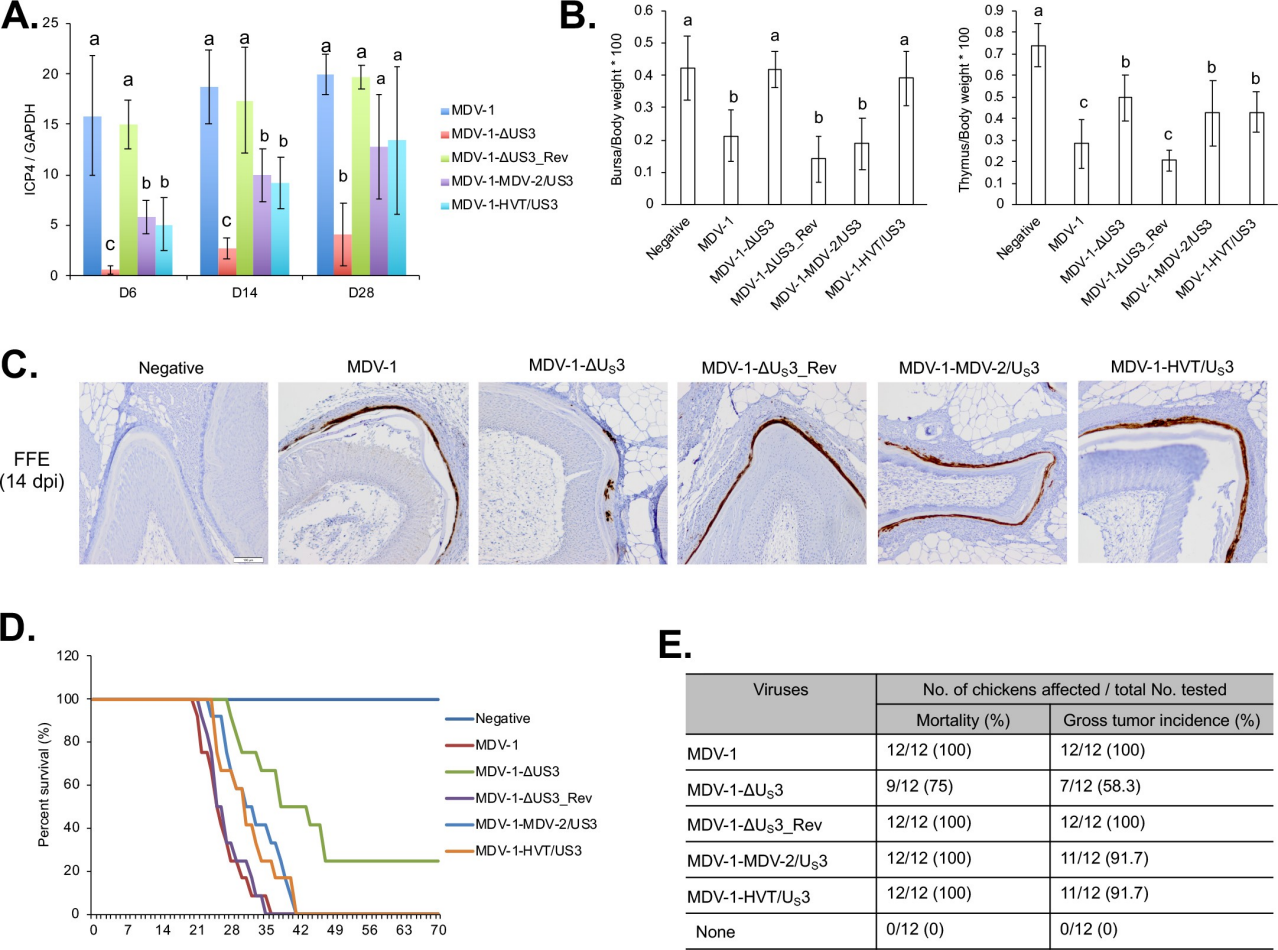
*In vivo* characterization of chimeric and revertant MDVs. (A) On 6, 14, and 28 days post inoculation (dpi), splenocytes isolated from 5 chickens infected with the indicated viruses were used for genomic DNA extraction. MDV genomic copy number was determined by qPCR. The relative MDV genomic copy number is presented as the average ratio of *ICP4* to *GAPDH* copy number with error bar representing the standard deviation. Significant difference between groups are marked as letters where different letters represent significant different at p<0.05. (B) On day 14 post inoculation, bursa, thymus, and body weights of 5 chickens infected with the indicated viruses or 5 negative control chickens were measured, and presented as ratio of bursa to body weight (left) and ratio of thymus to body weight (right). The graph represents the average ratio of 5 chickens with error bars showing the standard deviation. Significant difference between groups are marked as letters where different letters represent significant different at p<0.05. (C) At 14 dpi, feather follicle collected from 5 inoculated or negative control chickens were subjected to immunohistochemistry analysis using MDV pp38 antibody. Representative images were presented and scale bar = 100 μm. (D, E) After inoculating with the indicated viruses, chickens were maintained in isolation. The mortality of each group was recorded daily for 10 weeks and percent survival of chickens over time presented in the graph (D). Chickens that died during the experiment were examined for gross MDV specific tumors. The mortality rate and tumor incidence of each group are summarized (E).

To examine whether MDV-2 U_S_3 and HVT U_S_3 can rescue the replication deficiency of MDV-1 U_S_3 null virus *in vivo*, we examined the viral load in spleen of infected chickens during early cytolytic infection, latent infection, and late cytolytic infection stages. One-day-old chickens were inoculated subcutaneously with the different viruses. One group of chickens remained as uninoculated control. Five chickens from each group were randomly selected at 6, 14, and 28 days post inoculation, and spleens were collected for DNA isolation followed by qPCR to determine MDV genome copy number. Our results show that deletion of MDV-1 U_S_3 significantly reduces MDV replication in spleen at 6, 14, and 28 days post inoculation, which can be fully resorted in the revertant virus (MDV-1-ΔU_S_3_Rev) (Fig 2A). In addition, similar to *in vitro* results (Fig 1C), genome copy number of MDV-1-MDV-2/U_S_3 and MDV-1-HVT/U_S_3 was higher than MDV-1-ΔU_S_3 and lower than parental (MDV-1) and revertant (MDV-1-ΔU_S_3_Rev) viruses at 6, 14, and 28 days post inoculation (Fig 2A), indicating that MDV-2 U_S_3 and HVT U_S_3 can partially compensate the function of MDV-1 U_S_3 in MDV replication in splenocytes.

As the only site to produce fully infectious virus, the replication of MDV in FFE was studied by immunohistochemistry (IHC) assays using an MDV pp38 monoclonal antibody. Our results show that deletion of MDV-1 U_S_3 significantly impaired virus replication in the FFE, as indicated by low levels of MDV pp38 expression (Fig 2C). Interestingly, MDV-2 and HVT U_S_3 could fully compensate MDV replication in FFE, as levels of pp38 expression in FFE of MDV-1-MDV-2/U_S_3 and MDV-1-HVT/U_S_3 inoculated chickens were similar to those of chickens inoculated with parental and revertant viruses (Fig 2C). Taken together, our results prove that U_S_3 from MDV-2 and HVT can fully compensate MDV-1 U_S_3 functions in epithelial cells but not lymphocytes.

#### Lymphoid organ atrophy

Early cytolytic infection by MDV-1 causes lymphoid organ atrophy in chickens. Five chickens from each group were randomly selected at 14 days post inoculation, and lymphoid organ (bursa and thymus) to body weight ratios were calculated. Our results show that bursa/body weight ratio was significantly lower in parental MDV-1, MDV-1-ΔU_S_3_Rev, and MDV-1-MDV-2/U_S_3 infected chickens when compare to uninoculated chickens, while MDV-1-ΔU_S_3 and MDV-1-HVT/U_S_3 infection did not affect the bursa/body weight ratio (Fig 2B, left). Parental MDV-1 and MDV-1-ΔU_S_3_Rev infection also significantly reduced the thymus/body weight ratio (Fig 2B, right). In addition, the thymus/body weight ratio of MDV-1-ΔU_S_3, MDV-1-MDV-2/U_S_3, and MDV-1-HVT/U_S_3 infected chickens was significantly lower than uninoculated chickens but significantly higher than parental MDV-1 and MDV-1-ΔU_S_3_Rev infected chickens (Fig 2B, right). These results suggest that MDV-2 U_S_3 affects MDV-1 replication in both B- and T-cells, while HVT U_S_3 only affects MDV-1 replication in T-cells.

#### Survival rate and oncogenicity

To evaluate the pathogenicity of chimeric viruses, one-day-old chickens were inoculated subcutaneously and monitored for 10 weeks to evaluate MDV associated mortality and tumors. MDV associated mortality in parental MDV-1 and MDV-1-ΔU_S_3_Rev infected chickens started at 21–22 days post inoculation, all chickens died by day 34–35 post inoculation, and all chickens had gross MDV specific tumors (Fig 2D and 2E). On the other hand, MDV-1-MDV-2/U_S_3 and MDV-1-HVT/U_S_3 infected chickens started showing MDV associated mortality at 24–25 days post inoculation, all died by day 40 post inoculation, and 11 out of 12 chickens had gross MDV specific tumors (Fig 2D and 2E). Mortality in the MDV-1-ΔU_S_3 infected group started 28 days post inoculation, 9 out of 12 chickens died by the end of experiment, and only 7 out of 12 chickens had gross MDV specific tumors (Fig 2D and 2E). Uninoculated control group showed no MDV associated mortality or tumors. These results suggest that deletion of MDV-1 U_S_3 attenuate the virulence of MDV-1, which can be partially restored MDV-2 and HVT U_S_3.

### MDV U_S_3 phosphorylates chHDAC1 and 2

**Fig 3:**
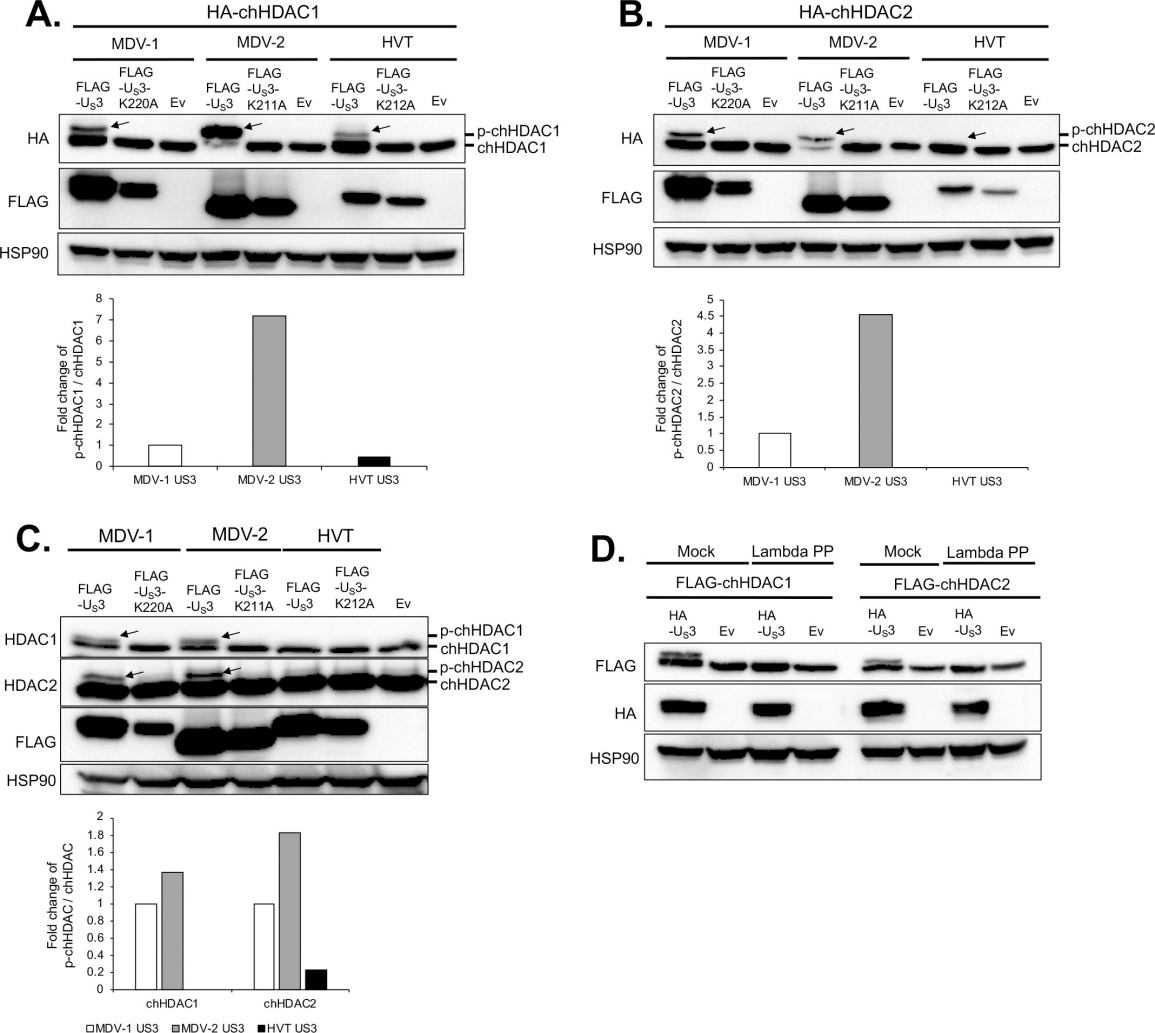
MDV U_S_3 mediates the phosphorylation of chHDAC1 and 2. pcDNA-HA-chHDAC1 (A) or pcDNA-HA-chHDAC2 (B) were co-transfected with pcDNA FLAG tagged wild type MDV-1, MDV-2, or HVT U_S_3, kinase dead U_S_3 (pcDNA-FLAG-U_S_3-K220A for MDV-1, pcDNA-FLAG-U_S_3-K211A for MDV-2, and pcDNA-FLAG-U_S_3-K212A for HVT), or pcDNA empty vector (Ev) to 293T cells. Forty-eight hours later, cells were lysed and subjected to Western blot (WB) analysis with FLAG antibody. HSP90 was stained as loading control. (C) DF-1 cells were transfected with the indicated plasmids for 48 hours, followed by WB with HDAC1, HDAC2, FLAG, and HSP90 antibodies. (D) pcDNA-FLAG-chHDAC1 (left) or pcDNA-FLAG-chHDAC2 (right) were co-transfected with pcDNA-HA-MDV-1-U_S_3 or pcDNA Ev into 293T cells for 48 hours. Whole cell lysates were either mock treated or treated with Lambda protein phosphatase (Lambda PP) prior to SDS-PAGE. WB was carried out with FLAG, HA, and HSP90 antibodies. Protein bands of phosphorylated chHDAC1 (p-chHDAC1) and p-chHDAC2 are marked by arrow. Phosphorylated and unmodified chHDAC1 or chHDAC2 protein levels were quantified with Image J and presented as fold changes of p-chHDAC to chHDAC ratio relative to MDV-1 U_S_3 transfected cells.

To study if U_S_3 of all three MDV types phosphorylates chHDAC1 and 2, we examined the electrophoretic mobility of chHDAC1 and 2 upon transfection of MDV U_S_3. As shown in Fig 3A, transfection of 293T cells with pcDNA expressing wild type U_S_3 from MDV-1, MDV-2, and HVT resulted in an additional slow migrating protein species of FLAG-chHDAC1 (marked by arrow) when compared to kinase dead U_S_3 (K220A for MDV-1 U_S_3, K211A for MDV-2 U_S_3, and K212A for HVT U_S_3) and empty vector (Ev) transfected cells. Interestingly, the amount of the higher molecular weight FLAG-chHDAC1 protein in wild type MDV-2 U_S_3 transfected cells was higher than in MDV-1 U_S_3 and HVT U_S_3 transfected cells (Fig 3A, bottom graph). Similar results were observed for chHDAC2, except that larger species of chHDAC2 could not be detected in wild type HVT U_S_3 transfected cells (Fig 3B). These results were further confirmed in co-transfected chicken embryonic fibroblasts (CEF) (S1A and S1B Fig) and chicken DF-1 cells (S1C and S1D Fig). Next, the electrophoretic mobility of endogenous chHDAC1 and 2 was examined in chicken DF-1 (Fig 3C) and CEF (S1E Fig) cells. After transfecting with pcDNA expressing wild type U_S_3, kinase dead U_S_3 or Ev, whole cell lysates were subjected to Western blot (WB) analysis with HDAC1 and 2 antibodies to specifically detect endogenous chHDAC1 and 2. Our results show that transfection of wild type MDV-1 and MDV-2 U_S_3, but not HVT U_S_3, led to two closely migrating chHDAC1 protein species (Figs 3C and S1E, first panel, marked by arrow). In addition, similar to Ev, transfection of kinase dead U_S_3 of all three MDV types did not result in modification of chHDAC1 as indicated by a single lower chHDAC1 protein band (Figs 3C and S1E, first panel). On the other hand, although transfection of wild type U_S_3, kinase dead U_S_3 or Ev resulted in two closely migrating chHDAC2 protein species, the amount of the larger molecular weight endogenous chHDAC2 was higher in wild type MDV-1 and MDV-2 U_S_3 transfected cells (Figs 3C and S1E, second panel, marked by arrow). Quantification analysis showed that ratios of p-chHDAC1/chHDAC1 and p-chHDAC2/chHDAC2 in wild type MDV-2 U_S_3 transfected cells were higher than in MDV-1 U_S_3 and HVT U_S_3 transfected cells (Fig 3C, bottom graph). To determine if the higher molecular weight species of chHDAC1 and 2 were the result of phosphorylation, we performed dephosphorylation assay using Lambda protein phosphatase (Lambda PP). Whole cell lysates extracted from HA tagged MDV-1 U_S_3 or Ev and FLAG-chHDAC1 or FLAG-chHDAC2 co-transfected 293T cells were either mock treated or treated with Lambda PP, followed by WB to examine their mobility in gels. In mock treated samples, transfection with wild type MDV-1 U_S_3 resulted in additional slow migrating protein species of chHDAC1 and 2, which were completely eliminated by Lambda PP treatment (Fig 3D), indicating MDV-1 U_S_3 induced modification of chHDAC1 and 2 is due to phosphorylation.

Considering that U_S_3 from all three MDV types showed distinct ability to phosphorylate chHDAC1 and 2, we investigated whether the same applies to other substrates. It has been shown previously that HSV-1 U_S_3 mimics the function of cellular protein kinase A (PKA) [22]. Here, we determined the ability of U_S_3 from all three MDV types to phosphorylate substrates of cellular protein kinases, including PKA, protein kinase C (PKC), and AMP-activated protein kinase (AMPK). Our results show that wild type MDV-1 and MDV-2 U_S_3 strongly increased the phosphorylation of PKA, PKC, and AMPK, while HVT U_S_3 only slightly increased their phosphorylation (S2 Fig).

### Identification of the chHDAC1 and 2 phosphorylation sites targeted by MDV U_S_3

**Fig 4:**
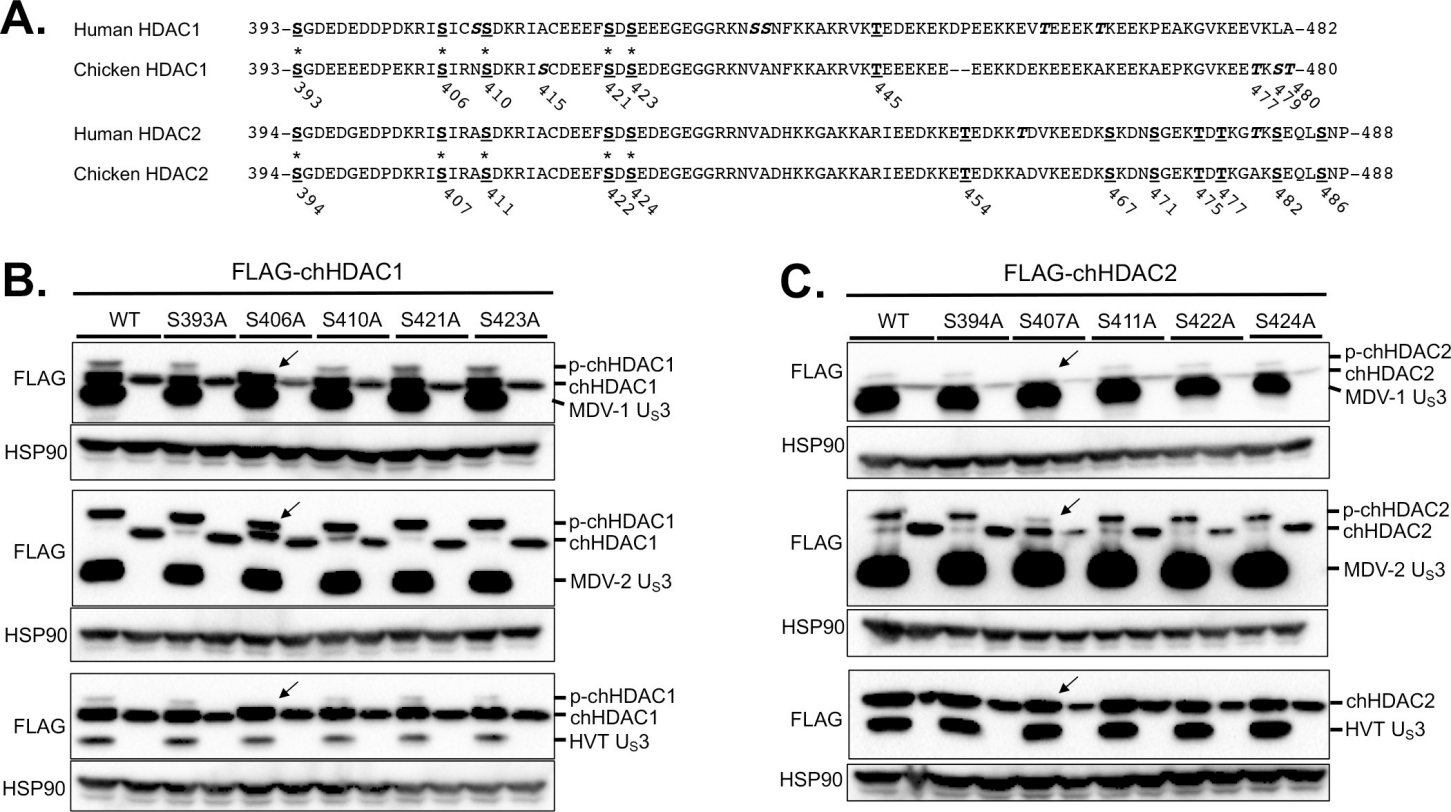
Identification of the phosphorylation sites in chHDAC1 and 2. (A) Amino acid sequence alignments of human HDAC1 and chicken HDAC1, and human HDAC2 and chicken HDAC2. Conserved serine (S) and threonine (T) sites are presented as bold underlined, and unique S and T sites are presented as bold italics (those marked with an * were mutated to alanine, A). The position of S and T in chicken HDAC1 and 2 are labeled below the amino acid sequences. pcDNA FLAG tagged MDV-1, MDV-2, or HVT U_S_3, or pcDNA empty vector were co-transfected with pcDNA-FLAG-chHDAC1 (B) or pcDNA-FLAG-chHDAC2 mutants (C) into 293T cells. Forty-eight hours later, cells were lysed and subjected to Western blot with FLAG and HSP90 antibodies. Protein bands of expected phosphorylated chHDAC1 (p-chHDAC1) and p-chHDAC2 are marked by black arrow.

It has been reported that HSV-1 U_S_3, VZV ORF66, and PRV U_S_3 phosphorylate HDAC1 at S406 and HDAC2 at S407 [16,18]. We were interested in identifying chHDAC1 and 2 phosphorylation sites targeted by MDV U_S_3. Because most post-translational modifications of HDAC1 and 2 happen at their C-terminal end, we first compared the C-terminal amino acid sequences of human HDAC1 and 2 with chicken HDAC1 and 2. Amino acid sequence alignments show that there are conserved serine (S) and threonine (T) sites (bold underlined) between human and chicken HDAC1 and 2, while there are also some unique S and T sites (bold italics) in chicken HDAC1 protein (Fig 4A).

Based on previous reports [16], we first mutated five conserved S sites (Fig 4A, marked with asterisk) in chHDAC1 and 2 to alanine (A). pcDNA expressing FLAG-chHDAC1 mutants were co-transfected with wild type MDV-1, MDV-2, HVT U_S_3, or Ev. After 48 hours, cells were lysed and subjected to WB to examine the electrophoretic mobility of chHDAC1 mutants. As shown in Fig 4B, mutation of S406A completely blocked chHDAC1 phosphorylation mediated by MDV-1 and HVT U_S_3 as the larger protein species was eliminated (marked by arrow), while other mutations, including S393A, S401A, S421A, and S423A, had no effect in MDV-1 and HVT U_S_3 induced chHDAC1 phosphorylation. Interestingly, we could still detect the chHDAC1 slow migrating protein species in the S406A mutant and MDV-2 U_S_3 co-transfected cells suggesting that MDV-2 U_S_3 has additional target sites (Fig 4B). For chHDAC2, we could not detect larger protein species in the S407A mutant and MDV-1 U_S_3 co-transfected cells, indicating that S407 of chHDAC2 is the only target site for MDV-1 U_S_3 (Fig 4C, marked by arrow). Similarly, MDV-2 U_S_3 has more targets in chHDAC2 as S407A mutation could not completely inhibit chHDAC2 phosphorylation (Fig 4C). Consistent with the above result (Fig 3B), HVT U_S_3 did not induce phosphorylation of chHDAC2 (Fig 4C).

**Fig 5:**
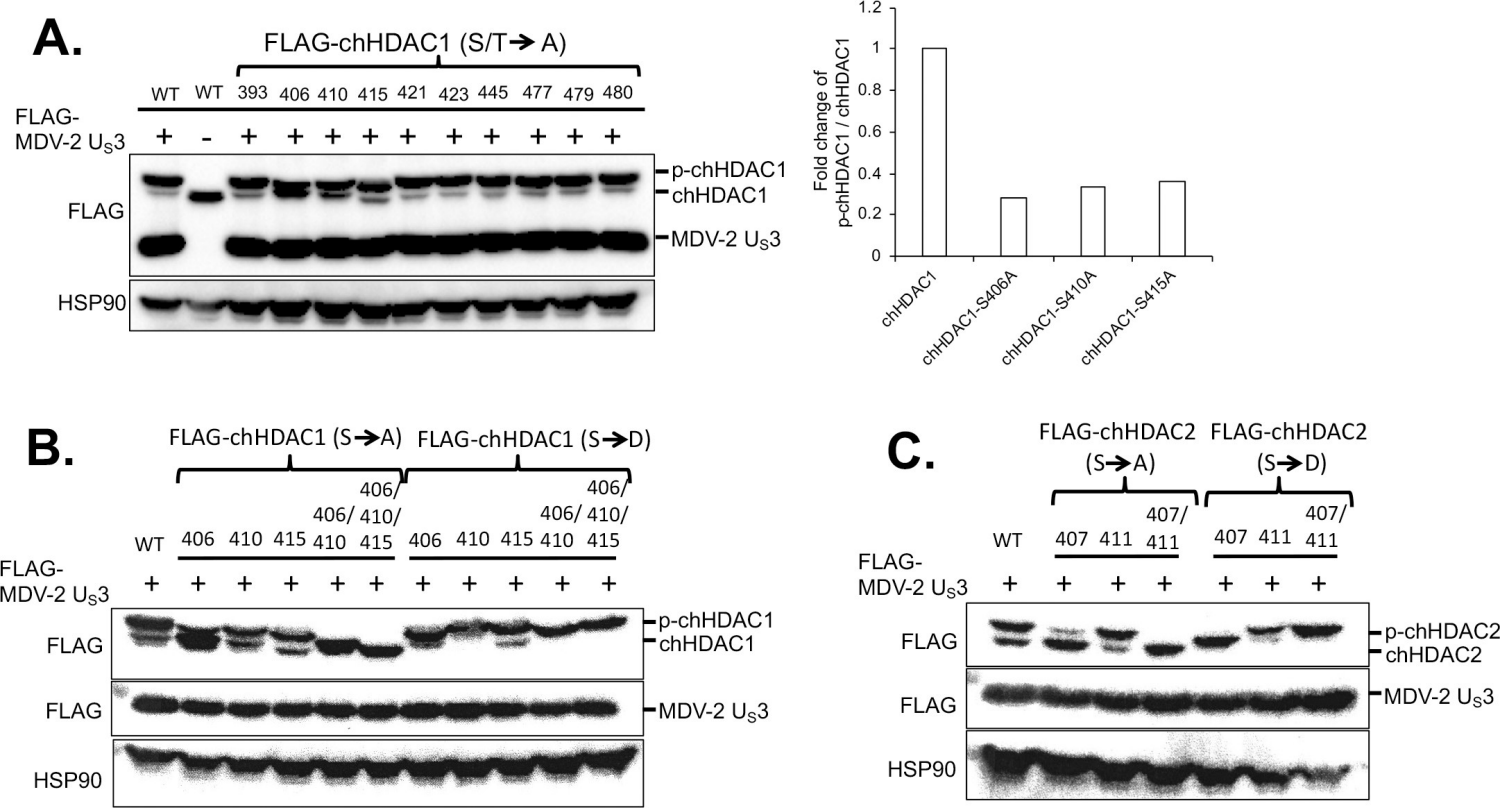
Mapping the MDV-2 U_S_3 target sites in chHDAC1 and 2. (A) pcDNA-FLAG-chHDAC1 single amino acid mutant plasmids was transfected with or without pcDNA-FLAG-MDV-2-U_S_3 into 293T cells for 48 hours, followed by Western blot (WB) with FLAG and HSP90 antibodies. Phosphorylated and unmodified mutant chHDAC1 protein levels were quantified with Image J and presented as fold changes of p-chHDAC1 to chHDAC1 ratio relative to MDV-2 U_S_3 and wild type chHDAC1 co-transfected cells. FLAG tagged single or multiple serine sites mutant chHDAC1 (B) or chHDAC2 (C) were co-transfected with pcDNA-FLAG-MDV-2-U_S_3 into 293T cells. Forty-eight hours later, WB was processed with FLAG antibody to examine the mobility of chHDAC1 or chHDAC2. HSP90 antibody was used as indicators of protein loading control.

Next, we mutated all other S and T sites in the C-terminus of chHDAC1 and examined the electrophoretic mobility of chHDAC1 mutants in MDV-2 U_S_3 co-transfected cells. WB (Fig 5A, left) and quantification (Fig 5A, right) analysis show that S406A, S410A, and S415A mutations resulted in higher amount of unmodified chHDAC1 (the smaller protein species) compared to wild type chHDAC1, indicating that MDV-2 U_S_3 may phosphorylate chHDAC1 at these three sites. These results were confirmed by the generation of double and triple chHDAC1 mutations where the triple mutant S406/410/415A completely eliminated MDV-2 U_S_3 mediated phosphorylation of chHDAC1 (Fig 5B). We further confirmed our results by examining the protein mobility of S to aspartic acid (D) mutant chHDAC1 (phosphorylation mimic form). As shown in Fig 5B, S406/410/415A triple mutant resulted in a single smallest protein species, while S406/410/415D triple mutant resulted in a single largest protein species. Using similar strategy, we showed that MDV-2 U_S_3 phosphorylates chHDAC2 at S407 and S411 residues (Fig 5C).

### Phosphorylation enhances the stability of chHDAC1, but not chHDAC2

**Fig 6:**
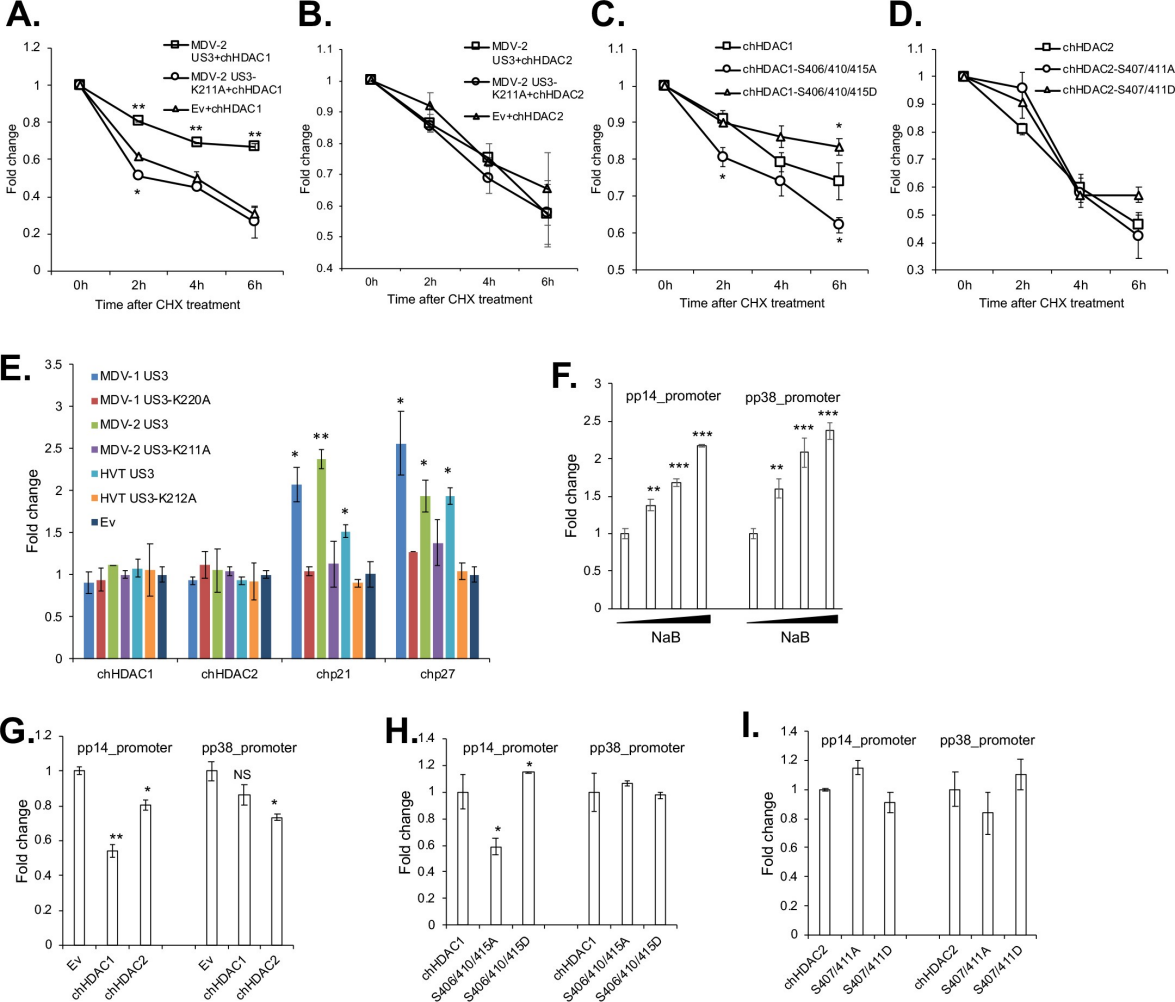
MDV U_S_3 induced phosphorylation regulates the stability and transcriptional regulation activity of chHDAC1 and 2. pcDNA-FLAG-chHDAC1 (A) or pcDNA-FLAG-chHDAC2 (B) was co-transfected with pcDNA expressing MDV-2 U_S_3, MDV-2-U_S_3-K211A, or empty vector (Ev) into 293T cells. Twenty-four hours later, transfected cells were treated with cycloheximide (CHX, 1 mg/ml) for 0-, 2-, 4-, or 6-hours. Then, cells were lysed and subjected to Western blot (WB) with FLAG and HSP90 antibodies. chHDAC1 or chHDAC2 protein levels were quantified with Image J, normalized to HSP90 protein level, and presented as fold changes relative to non-treated cells. pcDNA FLAG tagged wild type chHDAC1, chHDAC1-S406/410/415A mutant, or chHDAC1-S406/410/415D mutant (C); pcDNA FLAG tagged wild type chHDAC2, chHDAC2-S407/411A, or chHDAC2-S407/411D (D) were transfected into 293T cells. Twenty-four hours later, cells were treated with CHX (1 mg/ml) for 0-, 2-, 4-, or 6-hours. Then, cells were lysed and subjected to Western blot (WB) with FLAG and HSP90 antibodies. chHDAC1 or chHDAC2 protein levels were quantified with Image J, normalized to HSP90 protein level, and presented as fold change relative to non-treated cells. (E) DF-1 cells were transfected with wild type or kinase dead MDV-1, MDV-2, or HVT U_S_3, or empty vector (Ev). Forty-eight hours later, cells were harvest for RNA isolation followed by cDNA synthesis. qRT-PCR was carried out with the indicated primers. qRT-PCR data were analyzed by the 2^-ΔΔCT^ method using chicken *GAPDH* as internal control. Values are presented as fold change relative to Ev transfected cells. (F) 293T cells were transfected with pGL3-pp14_promoter or pGL3-pp38_promoter with *Renilla* luciferase vector. Twenty-four hours after transfection, cells were treated with different amount of sodium butyrate (NaB) overnight, followed by *Firefly* luciferase and *Renillla* luciferase activity measurement. Experiments were repeated two times in triplicate. Values are presented as fold change relative to non-treated cells. (G, H, I) The indicated plasmids were cotransfected with pGL3-pp14_promoter or pGL3-pp38_promoter and *Renilla* luciferase vector. Forty-eight hours later, cells were lysed with passive lysis buffer and processed as stated above. Values are presented as fold change relative to Ev (G), chHDAC1 (H) or chHDAC2 (I) transfected cells. NS: not significant, *: p<0.05, **: p<0.01, ***: p<0.001.

To discern the effect of MDV U_S_3 induced phosphorylation on chHDAC1 and 2, we examined the stability of phosphorylated and unmodified chHDAC1 and 2 in the presence of cycloheximide (CHX), a protein synthesis inhibitor. MDV-2 U_S_3 was used in this study because of the higher phosphorylation potential than MDV-1 and HVT U_S_3. Our results show that, after CHX treatment, chHDAC1 degrades slower in wild type MDV-2 U_S_3 and chHDAC1 co-transfected cells when compared to kinase dead MDV-2 U_S_3 or Ev and chHDAC1 co-transfected cells (Fig 6A). However, degradation of chHDAC2 was not affected by MDV-2 U_S_3 (Fig 6B). In addition, we found that chHDAC1-S406/410/415D mutant degrades slower than wild type chHDAC1 after CHX treatment, while chHDAC1-S406/410/415A mutant degrades faster, indicating that MDV U_S_3 induced phosphorylation stabilizes chHDAC1 protein (Fig 6C). On the other hand, degradation of chHDAC2-S407/411A and chHDAC2-S407/411D mutants was similar to wild type chHDAC2, suggesting that MDV U_S_3 induced phosphorylation does not affect the stability of chHDAC2 (Fig 6D).

### MDV U_S_3 induced phosphorylation regulates the transcriptional regulation activity of chHDAC1 and 2

To study the effect of phosphorylation in regulating chHDAC1 and 2 transcriptional regulation activity, we first examined the transcription of HDAC1 and 2 target genes, *p21* and *p27* [23,24], in MDV U_S_3 transfected cells. DF-1 cells were transfected with pcDNA expressing wild type or kinase dead MDV-1, MDV-2, and HVT U_S_3, or Ev. qRT-PCR analysis showed that wild type, but not kinase dead, U_S_3, from MDV-1, MDV-2 and HVT, significantly up-regulated the expression of chicken *p21* (*chp21*) and *chp27* (Fig 6E).

In addition, we found that treatment with sodium butyrate (NaB), an HDAC inhibitor, up-regulated the transcriptional activity of the well characterized MDV bidirectional *pp14* and *pp38* promoters [25], in a dose dependent manner (Fig 6F), suggesting that HDACs repress the transcriptional activity of *pp14* and *pp38* promoters. Next, we specifically examined the role of chHDAC1 and 2 in regulating the transcriptional activity of *pp14* and *pp38* promoters. Our results show that chHDAC1 significantly represses the transcriptional activity of *pp14* promoter (~1.8 fold reduction), but not *pp38* promoter (~1.2 fold reduction), while chHDCA2 suppresses the activity of both promoters (Fig 6G). In addition, compared to wild type chHDAC1, chHDAC1-S406/410/415A mutant represses the transcriptional activity of *pp14* promoter (~1.7 fold reduction), while chHDAC1-S406/410/415D mutant activates the transcriptional activity of *pp14* promoter (~1.2 fold activation), indicating that MDV induced phosphorylation inhibit the repressive effect of chHDAC1 on the *pp14* promoter (Fig 6H). As expected, wild type chHDAC1, chHDAC1-S406/410/415A and chHDAC1-S406/410/415D had no effect on the transcriptional activity of *pp38* promoter (Fig 6H). Results also show that MDV U_S_3 induced phosphorylation of chHDAC2 did not affect its role in regulating *pp14* and *pp38* promoter activity as chHDAC2-S407/411A and chHDAC2-S407/411D performed similar to wild type chHDAC2 in the dual luciferase assay (Fig 6I).

### MDV U_S_3 induced chHDAC1 and 2 phosphorylation regulates their interactions

**Fig 7:**
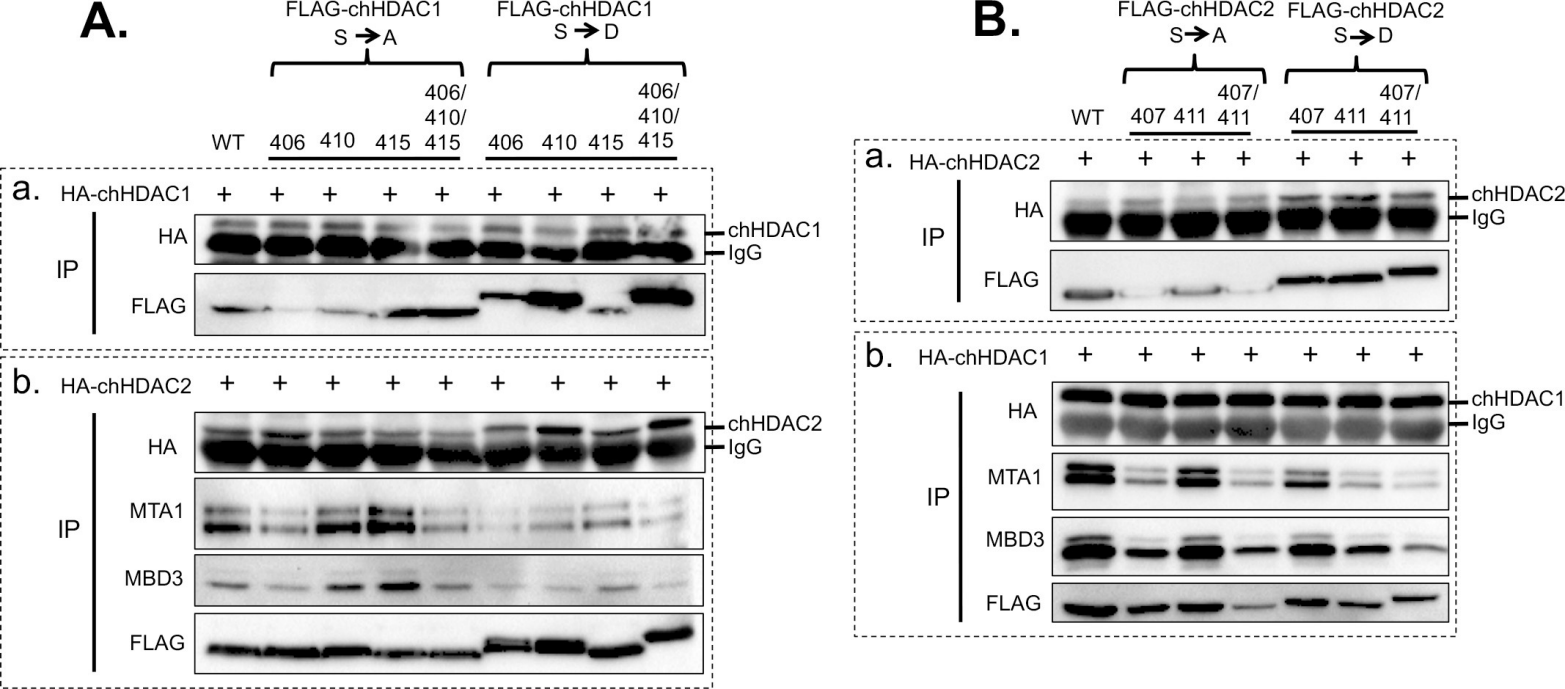
MDV U_S_3 induced chHDAC1 and 2 phosphorylation regulates their interactions. (A) pcDNA FLAG tagged wild type chHDAC1 or mutant chHDAC1 were co-transfected with pcDNA-HA-chHDAC1 (a) or pcDNA-HA-chHDAC2 (b) into 293T cells. Forty-eight hours later, cells were lysed and subjected to immunoprecipitation (IP) followed by Western blot (WB) analysis with the indicated antibodies. (B) pcDNA FLAG tagged wild type chHDAC2 or mutant chHDAC2 were transfected with pcDNA-HA-chHDAC2 (a) or pcDNA-HA-chHDAC1 (b). IP and WB were processed as stated above.

HDAC1 and 2 form homodimers and heterodimers through their N-terminal dimerization domain, and are components of repressor protein complexes, including CoREST, Sin3, and NuRD [11]. Here, we examined the effect of MDV U_S_3 induced phosphorylation in regulating chHDAC1 and 2 homodimerization, heterodimerization, and interaction with other proteins. As shown in Fig 7A, pcDNA expressing FLAG-chHDCA1 phosphorylation site mutants were co-transfected with HA-chHDAC1 (Fig 7A(a)) or HA-chHDAC2 (Fig 7A(b)) into 293T cells followed by immunoprecipitation (IP) and WB. Our results show that mutation of chHDCA1 phosphorylation sites did not affect their interaction with HA-chHDAC1 (Fig 7A (a)), indicating that MDV U_S_3 induced chHDAC1 phosphorylation has no effect on chHDAC1 homodimerization. On the other hand, compared to wild type chHDAC1, chHDAC1-S406/410/415A interacted weakly with HA-chHDAC2 while chHDAC1-S410D and chHDAC1-S406/410/415D showed strong interaction with HA-chHDAC2, indicating that MDV U_S_3 induced phosphorylation enhances the affinity between chHDAC1 and chHDCA2 (Fig 7A (b)). We also studied the interactions between chHDAC1 mutants and NuRD protein complex. Our results show that chHDAC1-S406A, chHDAC1-S406/410/415A, and all chHDAC1 S to D mutants exhibit lower affinity for MTA1 and MBD3 when compared to wild type chHDAC1 (Fig 7A (b)), suggesting that MDV U_S_3 induced phosphorylation affects the interactions between chHDAC1 and its cellular partners.

Next, similar experiments were performed to determine whether MDV U_S_3 induced chHDAC2 phosphorylation would affect the chHDAC2 interaction network. Our results show that chHDAC2 S to A mutations did not affect its homodimerization ability while chHDAC2 S to D mutants exhibit stronger interaction with wild type chHDAC2 (Fig 7B (a)). However, none of the chHDAC2 mutants showed any difference in their interactions with chHDAC1 (Fig 7B (b)), suggesting that MDV U_S_3 induced chHDAC2 phosphorylation enhances its homodimerization but does not affect its heterodimerization. The interaction between chHDAC2 mutants and cellular interaction partners were also examined. Compared to wild type chHDAC2, chHDAC2 S411A and S407D mutants showed similar levels of association with MTA1 and MBD3, while S407A, S407/411A, S411D, and S407/411D clearly showed less interaction with MTA1 and MBD3 (Fig 7B (b)), suggesting that MDV U_S_3 induced phosphorylation of chHDAC2 also affects its interactions with cellular partners.

### MDV U_S_3 interacts with chHDAC1 and 2

**Fig 8:**
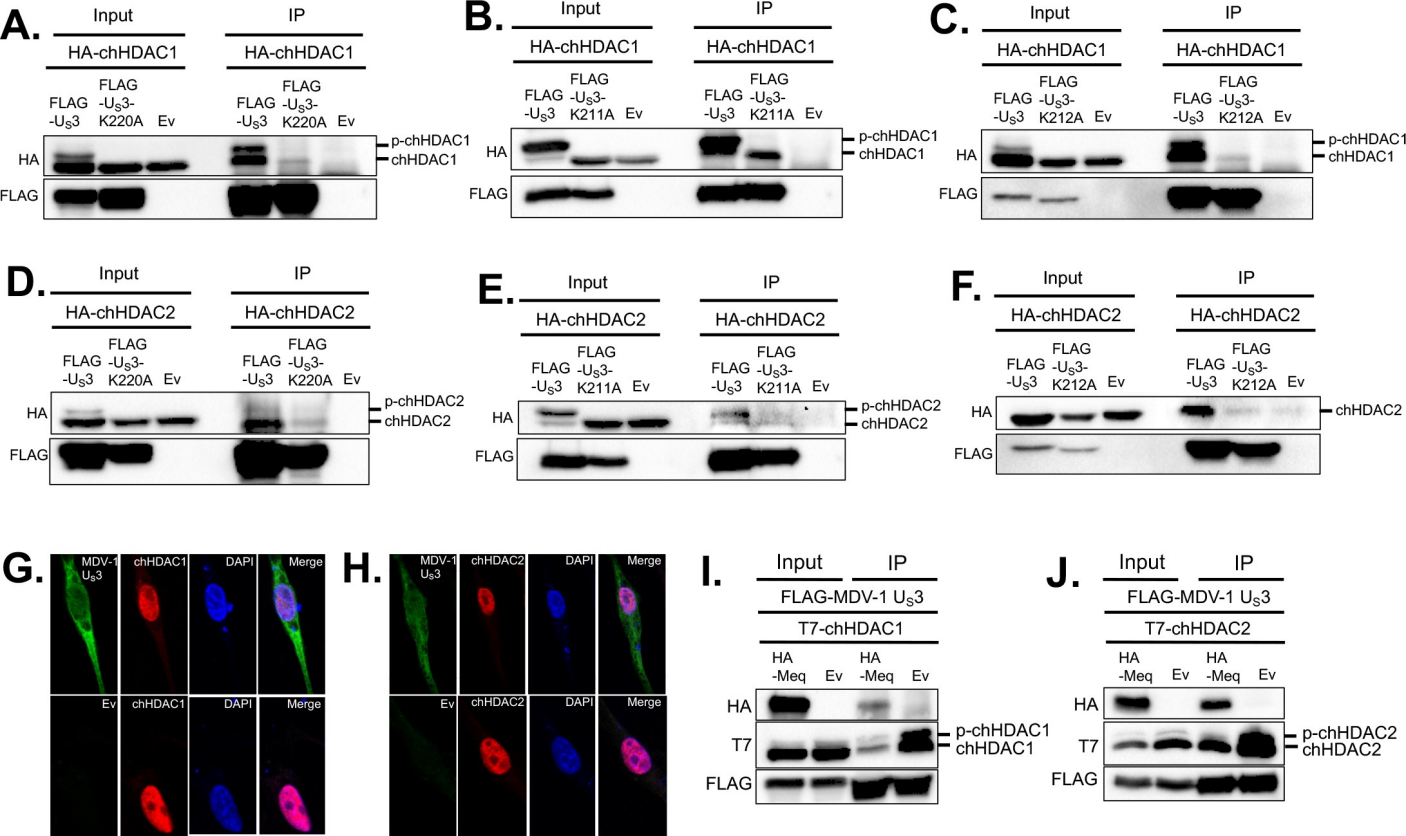
MDV U_S_3 interacts with chHDAC1 and 2. pcDNA-FLAG-MDV-1-U_S_3, pcDNA-FLAG-MDV-1-U_S_3-K220A, or pcDNA empty vector (Ev) were co-transfected with pcDNA-HA-chHDAC1 (A) or pcDNA-HA-chHDAC2 (D) into 293T cells. After 48 hours, cells were lysed and subjected to immunoprecipitation (IP). Western blot (WB) analysis was performed with HA and FLAG antibodies. The interactions between MDV-2 U_S_3 and chHDAC1 (B) or chHDAC2 (E), as well as HVT U_S_3 and chHDAC1 (C) or chHDAC2 (F) were determined by a similar method. DF-1 cells were transfected with pcDNA-FLAG-MDV-1-U_S_3 or Ev. Forty-eight hours later, cells were fixed for immunofluorescence assay with FLAG and HDAC1 (G) or HDAC2 (H) antibodies. DAPI was used to stain cell nuclei. All images were recorded using a confocal microscope. pcDNA-FLAG-MDV-1-U_S_3 was co-transfected with pcDNA-HA-Meq or pcDNA Ev, as well as pcDNA-T7-chHDAC1 (I) or pcDNA-T7-chHDAC2 (J) into 293T cells. Forty-eight hours later, IP was performed with mouse anti-FLAG agarose beads, followed by WB with HA, T7 and FLAG antibodies.

Apart from phosphorylation, we found that MDV-1 U_S_3 interacts with chHDAC1 and 2. Plasmids expressing FLAG tagged wild type MDV-1 U_S_3, kinase dead (MDV-1 U_S_3-K220A) U_S_3, or Ev were co-transfected with plasmids expressing HA-chHDAC1 or HA-chHDAC2, into 293T cells. Forty-eight hours later, whole cell lysates were subjected to IP with mouse anti-FLAG agarose beads followed by WB analysis. As shown in Fig 8A, wild type MDV-1 U_S_3 efficiently co-precipitated unmodified chHDAC1 and phosphorylated chHDAC1, while MDV-1 U_S_3-K220A only weakly co-precipitated unmodified chHDAC1, indicating that MDV-1 U_S_3 interacts with chHDAC1 via a kinase activity dependent manner. These results were further confirmed in co-transfected CEF (S3G Fig) and DF-1 cells (S3H Fig). Similar interaction pattern was observed between MDV-1 U_S_3 and chHDAC2 (Fig 8D). We also examined interaction between U_S_3 of MDV-2 and HVT, and chHDAC1 and 2. Our results show that wild type MDV-2 U_S_3 associates with unmodified and phosphorylated chHDAC1 and 2, while MDV-2 U_S_3-K211A (kinase dead) only weakly interacts with unmodified chHDAC1 and 2 (Fig 8B–8E). Similar results were found for HVT U_S_3 (Fig 8C–8F), with the exception that HVT U_S_3 failed to phosphorylate chHDAC2, as stated above. Same results were observed in reverse experiments where IP was performed to pull-down FLAG tagged chHDAC1 or chHDAC2 and WB was performed to detect HA tagged wild type U_S_3 or kinase dead U_S_3 (S3A–S3F Fig).

Using confocal microscopy, we also examined the subcellular co-localization of MDV-1 U_S_3 and endogenous chHDAC1 and 2. As expected, MDV-1 U_S_3 distributed throughout the cell, while chHDAC1 and 2 localized in the nucleus (Fig 8G and 8H). Our results also suggest that nuclear MDV-1 U_S_3 co-localizes with chHDAC1 (Fig 8G, upper panel) and chHDAC2 (Fig 8H, upper panel) in the nucleus of MDV-1 U_S_3 transfected DF-1 cells. In addition, unlike HSV-1 U_S_3 [17], transfection of MDV-1 U_S_3 did not affect the subcellular localization and distribution of chHDAC1 (Fig 8G) and chHDAC2 (Fig 8H) when compared to Ev transfected cells.

We have recently demonstrated that MDV-1 U_S_3 interacts with MDV Meq oncoprotein [9]. Here we also examined whether the presence of Meq affects the interaction between MDV-1 U_S_3 and chHDAC1 and 2. IP results show that FLAG tagged MDV-1 U_S_3 could coprecipitate Meq as well as unmodified and phosphorylated chHDAC1 (Fig 8I) or chHDAC2 (Fig 8J). We also observed that levels of chHDAC1 and 2 proteins were lower in the presence of Meq in both input and IP samples, which is most likely due to Meq mediated degradation of chHDAC1 and 2. These results suggest that Meq has no effect on the association between MDV-1 U_S_3 and chHDAC1 and 2.

### HDAC activity affects replication and plaque size of MDV-1

**Fig 9:**
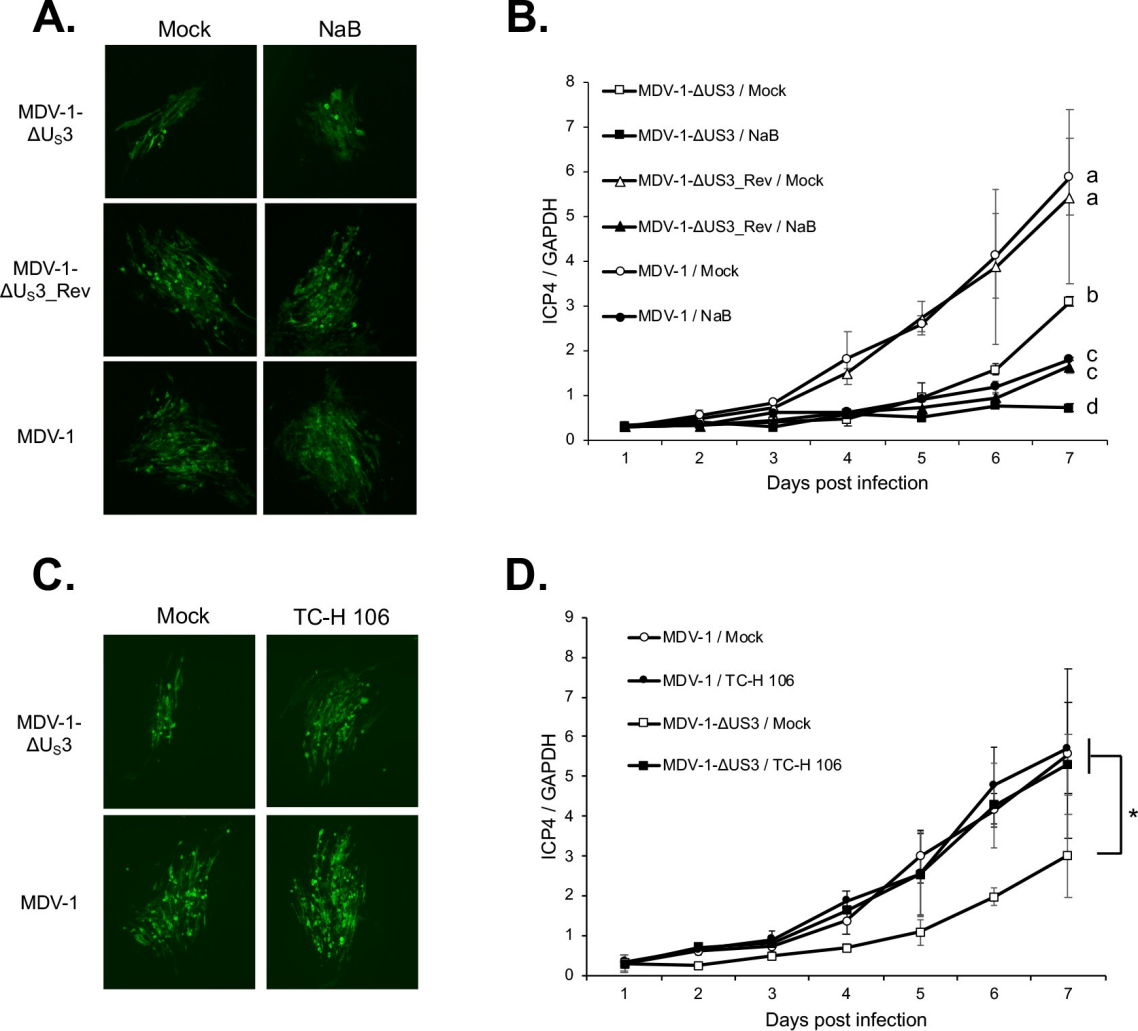
Effects of HDAC inhibitors to the plaque size and replication of MDV-1. Mock and NaB (1 mM) treated (A) or mock and TC-H 106 (10 μM) (C) treated chicken embryonic fibroblasts (CEF) were infected with MDV-1-ΔU_S_3, MDV-1-ΔU_S_3_Rev, or parental MDV-1 viruses, respectively. Seven days later, cells were fixed and subjected to immunofluorescence assay with MDV pp38 mouse monoclonal antibody and goat anti-mouse Alexa Fluor 488. All images were taken with a 10x objective. Mock and NaB (1 mM) treated (B) or mock and TC-H 106 (10 μM) (D) treated CEF was infected with MDV-1-ΔU_S_3, MDV-1-ΔU_S_3_Rev, or parental MDV-1 viruses, respectively. Cells were harvested daily until 7 days post infection, followed by genomic DNA isolation. Viral genome copy number was determined by qPCR using MDV *ICP4* and chicken *GAPDH* primers, and presented as the ratio of *ICP4* to *GAPDH* copy number with error bar representing the standard deviation. Significant difference between groups are marked as * or letters where different letters represent significant different at p<0.05.

It has been reported that inhibition of HDAC activity by sodium butyrate (NaB) treatment enhances growth, plaquing efficiency, and plaque size of VZV ORF66 null virus but not parental virus [16]. In addition, NaB treatment enhanced plaquing efficiency of PRV U_S_3 null virus but not HSV-1 U_S_3 null virus [18]. To explore the role of chHDAC1 and 2 in regulating MDV replication, we examined the replication and plaque size of parental MDV-1, U_S_3 deletion MDV-1 (MDV-1-ΔU_S_3), and U_S_3 deletion revertant MDV-1 (MDV-1-ΔU_S_3_Rev) in NaB treated CEF. Our results show that treatment with NaB did not affect the plaque size of parental MDV-1, MDV-1-ΔU_S_3, and MDV-1-ΔU_S_3_Rev (Fig 9A). Surprisingly, the replication of MDV-1, MDV-1-ΔU_S_3, and MDV-1-ΔU_S_3_Rev in CEF, as measured by MDV genome copy number, was suppressed by treatment with NaB (Fig 9B).

Since NaB inhibits the activity of both class I and class II HDACs, we repeated the experiments using pimelic diphenylamide 106 (TC-H 106), which specifically inhibits the activity of class I HDACs. Our results show that both plaque size (Fig 9C) and genome replication (Fig 9D) of MDV-1-ΔU_S_3 virus were enhanced by TC-H 106 treatment to a level comparable with parental MDV-1, indicating that MDV-1 U_S_3 mediated modifications of chHDAC1 and 2 plays a critical role in the regulation of MDV plaque size and genome replication. Taken together, these results suggest that class I HDACs suppress MDV plaque size and replication, while the enzymatic activity of class II HDACs is important for efficient MDV replication.

## Discussion

Herpesvirus life cycle consists of distinct lytic and latent phases. Upon infection with a herpesvirus, host factors rapidly respond to silence viral gene expression to further limit viral replication. A well-known group of host repressor is the HDAC enzyme family which mediate the removal of acetyl molecules from modified lysine residues and subsequently represses gene expression. HDACs regulate herpesviruses infection and in turn herpesviruses have evolved mechanisms to modulate HDACs repressor complexes. One of the strategies utilized by herpesviruses is through virus-host protein interactions. HSV-1 ICP0 has been shown to associate with HDAC1 to interrupt CoREST complex, and Kaposi’s sarcoma-associated herpesvirus (KSHV) K-bZIP has been reported to interact with HDAC2 to recruit it to viral promoters [17,26]. A second strategy is through post-translational modification. Walters *et al*. have identified HDAC1 and 2 as conserved targets of VZV, HSV-1, and PRV U_S_3 orthologs [16,18]. HSV-1 U_S_3 protein kinase phosphorylates HDAC1 and 2 to promote efficient viral gene expression and genome replication [17]. Other studies further demonstrated that VZV ORF66 and PRV U_S_3 exhibit similar function by phosphorylating HDAC1 and 2 [16,18]. Until this study, the role of MDV U_S_3 in regulating HDAC1 and 2 phosphorylation and function was not known.

To dissect the role of MDV U_S_3 in MDV replication and pathogenesis, we constructed chimeric viruses by replacing U_S_3 of MDV-1 with that of MDV-2 and HVT. First, we observed that deletion of MDV-1 U_S_3 resulted in smaller plaque size (Fig 1A), lower plaquing efficiency (Fig 1B), and genome replication (Fig 1C), suggesting that MDV-1 U_S_3 is involved in the replication of viral genome as well as MDV plaque forming efficiency. In addition, we characterized the chimeric viruses *in vitro* and our results show that MDV-2 and HVT U_S_3 could partially rescue the plaque size and growth of MDV-1-ΔU_S_3 virus (Fig 1). Similarly, we found MDV-1 U_S_3 deletion resulted in growth deficiency of MDV-1-ΔU_S_3 in splenocytes of infected chickens, which can be partially recovered by MDV-2 and HVT U_S_3 (Fig 2A). In addition, replication of MDV-1 in FFE was also impaired by deletion of MDV-1 U_S_3 (Fig 2C), indicating the transmission of MDV-1-ΔU_S_3 virus may be impaired. However, the growth deficiency of MDV-1-ΔU_S_3 in FFE was fully restored by MDV-2 and HVT U_S_3 (Fig 2C). We also analyzed lymphoid organ atrophy in chimeric viruses infected chickens. Infection of parental and revertant MDV-1 resulted in severe bursa atrophy, which could be fully eliminated by MDV-1 U_S_3 deletion and HVT U_S_3 replacement, but not MDV-2 U_S_3 replacement. On the other hand, thymus atrophy induced by infection with parental and revertant MDV-1 could be partially reverted by MDV-1 U_S_3 deletion, and replacement with MDV-2 and HVT U_S_3 (Fig 2B). Although the exact mechanisms behind this phenotype need further study, we speculate that MDV-2 U_S_3 does not affect the replication of chimeric virus in B-cells leading to severe bursa atrophy, while HVT U_S_3 largely decreased the replication of chimeric virus in B-cells that led to no bursa atrophy while both MDV-2 and HVT U_S_3 partially decreased the replication of chimeric viruses in T-cells leading to mild thymus atrophy. These results suggest that deletion of MDV-1 U_S_3, and expression of MDV-2 and HVT U_S_3 attenuated MDV-1 differently to reduce lymphoid organ atrophy. This conclusion was confirmed in chimeric viruses infected chickens in which deletion of U_S_3, and expression of MDV-2 and HVT U_S_3 delayed the MDV associated death of infected chickens and reduced MDV specific tumor incidence (Fig 2D and 2E). Next generation sequencing of splenocytes isolated from chickens infected with chimeric viruses, at different infection stages, will help investigate cellular genes and pathways that are differentially regulated by U_S_3 from all three MDV types.

To investigate if MDV U_S_3 phosphorylates HDAC1 and 2, and the potential effect of this phosphorylation. Since all three MDV types encode a U_S_3 protein kinase which share a conserved kinase activity domain [9] we first analyzed the role U_S_3 from all three MDV types in chHDAC1 and 2 phosphorylation. Our findings show that U_S_3 from MDV-1 and MDV-2 phosphorylates both chHDAC1 and 2, while HVT U_S_3 only phosphorylates chHDAC1 (Figs 3 and S1). Interestingly, we observed that MDV-2 U_S_3 exhibits stronger capacity to phosphorylate chHDAC1 and 2 compared to MDV-1 and HVT U_S_3 (Figs 3 and S1). These findings demonstrate that MDV U_S_3 shares the ability of HSV-1 U_S_3, VZV ORF66, and PRV U_S_3 to phosphorylate chHDAC1 and 2. We further investigated the MDV U_S_3 target sites in chHDAC1 and 2 and identified novel phosphorylation sites in both proteins. Specifically, our results show that MDV-1 and HVT U_S_3 phosphorylate chHDAC1 at S406 and MDV-2 phosphorylates chHDAC1 at S406, S410, and S415; MDV-1 phosphorylates chHDAC2 at S407 and MDV-2 phosphorylates chHDAC1 at S407 and S411 (Figs 4 and 5). We speculate that this phosphorylation is through an indirect pathway as previous studies showed that HDAC2 is an indirect substrate of VZV ORF66 [16]. CKII has been identified as the main upstream kinase of HDAC1 and 2, and HDAC1, but not HDAC2, was shown to be a substrate of protein kinase A (PKA) [11]. Although our results show that MDV U_S_3 mimics the function of cellular PKA, PKC, and AMPK to increase phosphorylation of their substrates (S2 Fig), further studies are needed to identify cellular kinases involved in MDV U_S_3 mediated phosphorylation of chHDAC1 and 2. In addition, the mechanism behind the differential phosphorylation capacity of U_S_3 from all three MDV types needs further studies. Protein sequence alignment and 3D structure predictions show that the catalytic active site containing region of U_S_3 from all three MDV types is highly conserved; however, the N terminal 100 amino acids of MDV-1, MDV-2, and HVT U_S_3 are highly variable. We hypothesize that MDV-1, MDV-2, and HVT U_S_3 may associate with different host factors or manipulate different cellular signaling pathways through the N-terminal variable region to exhibit distinguishing functions. Next generation sequencing and proteomic methods will help to comprehensively study the interplay between MDV U_S_3 and host factors.

Although HDAC1 and 2 have been identified as the substrates of alphaherpesviruses U_S_3, the effect of U_S_3 mediated phosphorylation on the biochemical properties of HDAC1 and 2 have not yet been studied. Here, we investigated the effect of MDV U_S_3 induced phosphorylation on the functions of chHDAC1 and 2. Our results show that MDV-2 induced phosphorylation enhanced the stability of chHDAC1 but does not affect the stability of chHDAC2 (Fig 6A–6D). In addition, expression of chHDAC1 and 2 target genes, *chp21* and *chp27*, were also up-regulated in response to overexpression of MDV-1, MDV-2, and HVT U_S_3 (Fig 6E), suggesting that MDV U_S_3 induced phosphorylation inhibits the transcriptional repressive function of chHDAC1 and 2 to allow for efficient gene expression. p21 and p27 are well characterized CIP/Kip family of cyclin-dependent kinase (CDK) inhibitors which are involved in cell cycle regulation and apoptosis inhibition [27,28]. We speculate that MDV U_S_3 induced *chp21* and *chp27* expression contributes to its apoptosis inhibition function as previously reported [6,29,30]. Furthermore, the up-regulated expression of *chp21* and *chp27*, in response to transfection of MDV U_S_3, suggests that MDV U_S_3 may function in cell cycle regulation. We also found that S406/410/415 phosphorylation of chHDAC1 weakens its repressive effect on the MDV *pp14* promoter but not on the MDV *pp38* promoter, while S407/411 phosphorylation of chHDAC2 had no effect on its repressive effect of MDV *pp14* and *pp38* promoters (Fig 6F–6I). These results suggest that chHDAC1 and 2 regulate the transcription of MDV genes in both phosphorylation dependent and independent manners. Besides transcriptional regulation, the interaction networks of chHDAC1 and 2 were also affected by MDV U_S_3 mediated phosphorylation (Fig 7), similar to CKII induced phosphorylation [15]. Further, we found that MDV U_S_3 associates with chHDAC1 and 2 in a phosphorylation dependent manner (Figs 8 and S3), suggesting MDV U_S_3 may also regulate the function of chHDAC1 and 2 through interactions other than phosphorylation.

In contrast to a previous report by Walters *et al*. [16], who described that sodium butyrate (NaB) treatment increased the plaquing efficiency, plaque size, and growth of VZV ORF66 null virus but not wild type virus in MeWo cells, our results show that NaB treatment, which inhibit the enzymatic activity of both class I and class II HDACs, did not affect the plaque size of MDV-1 U_S_3 null (MDV-1-ΔU_S_3) virus but inhibited the growth of MDV-1-ΔU_S_3 and parental MDV-1 viruses (Fig 9A and 9B). However, we further found that treatment with class I HDACs specific inhibitor, TC-H 106, increased the plaque size (Fig 9C) and genome replication (Fig 9D) of MDV-1-ΔU_S_3 virus to a level comparable with parental MDV-1. These results suggest that class I HDACs and class II HDACs differentially regulate MDV-1 plaque size and replication, and strongly indicate that the MDV-1 U_S_3 mediated manipulation of chHDAC1 and 2 contributed to the plaque size and genome replication of MDV-1. Since the replication of both MDV-1-ΔU_S_3 and parental MDV-1 viruses was inhibited by NaB treatment (Fig 9B), we hypothesize there are other viral factors, such as MDV-1 UL13 protein kinase, that also target chHDACs to facilitate the replication of MDV-1 since it has been reported that mouse gammaherpesvirus 68 (MHV68) ORF36 protein kinase (an HSV-1 UL13 orthologous), interacts with HDAC1 and 2 to promote viral replication [31]. It is possible that MDV-1 UL13 share this function and, in combination with MDV-1 U_S_3, targets chHDACs to regulate MDV-1 replication. In addition, MDV-1 U_S_3 may also target other cellular and viral proteins to regulate MDV-1 replication. We have previously showed that MDV-1 U_S_3 phosphorylates CREB to regulate expression of cellular and viral genes [32], which may also contribute to the replication of MDV-1. Given the multifunctional property of U_S_3 protein kinase, it is possible that MDV-1 U_S_3 regulates cellular activators (e.g. CREB) and repressors (e.g. HDAC 1 and 2) functions to fulfill the MDV life cycle. In addition, since NaB and TC-H106 treatment inhibits class I and class II HDACs or class I HDACs, respectively [33], it is difficult to distinguish the role of individual HDAC in regulating herpesvirus replication. Further studies using knockout methods, such as short hairpin RNA (shRNA) and clustered regularly interspaced short palindromic repeats (CRISPR), that specifically target individual HDAC will help reveal the specific function of each HDAC in regulating herpesvirus gene expression and replication.

In conclusion, using a natural MD chicken model, our studies provide the first *in vivo* evidence on the role of MDV-1 U_S_3 in MDV-1 replication and pathogenesis, and demonstrate that U_S_3 from MDV-2 and HVT can partially compensate the functions of MDV-1 U_S_3 *in vitro* and *in vivo*. Our study also identified chHDAC1 and 2 as novel substrates and interaction partners of MDV U_S_3, and determined novel phosphorylation sites in chHDAC1 and 2. We demonstrate that U_S_3 of MDV and HVT function differently in phosphorylating chHDAC1 and 2. Biochemical analysis further characterized the effect of MDV and HVT U_S_3 induced phosphorylation in regulating functions of chHDAC1 and 2. With the application of HDAC inhibitors, we showed the interplay between MDV-1 U_S_3 and chHDAC1 and 2 in the regulation of MDV-1 plaque size and genome replication. The interaction between herpesvirus U_S_3 protein kinase and HDACs studied in a natural virus-host model presented here will have a broader impact on our understanding of herpesvirus biology.

## Materials and methods

### Ethics statement

All animal experiments were conducted following protocols approved by the Texas A&M University Institutional Animal Care and Use Committee (IACUC).

### Cell culture

10–11 day-old chicken embryos were used to prepare chicken embryonic fibroblasts (CEF) [34]. CEF were maintained in Leibowitz–McCoy (LM, 1:1) medium supplemented with 5% newborn calf serum. Chicken DF-1 cells and human embryonic kidney 293T cells were maintained in Dulbecco’s modified Eagle medium (DMEM) supplemented with 10% fetal bovine serum. All cells were maintained at 37°C in the presence of 5% CO_2._ CEF were used for recombinant virus production, virus titration and MDV *in vitro* growth kinetics assay. DF-1 and 293T cells were used for transient transfections.

### Mutagenesis of MDV-1 bacterial artificial chromosome (BAC)

Construction of MDV-1-ΔU_S_3 virus was described previously [9]. All primers used for mutagenesis of MDV-1 BAC are listed in S1 Table. To generate a chimeric MDV-1 virus expressing MDV-2 U_S_3 (MDV-1-MDV-2/U_S_3) BAC, MDV-2 U_S_3 ORF was amplified using MDV-2/U_S_3_F and MDV-2/U_S_3_R primers, and cloned into pUC19 plasmid to generate pUC19- MDV-2/U_S_3. Then, *Kan*^R^ gene was amplified with primers MDV-2/U_S_3Kan-F and MDV-2/U_S_3Kan-R with pEPKan-S plasmid as the template. The amplified product was digested and cloned into *EcoR*V site of pUC19-MDV-2/U_S_3 to generate pUC19-MDV-2/U_S_3-Kan. Next, the MDV-2 U_S_3 with *Kan*^R^ insertion was amplified with primers MDV-2/U_S_3-T-F and MDV-2/U_S_3-T-R to generate MDV-2/Us3-Kan transfer cassette that was transformed by electroporation into competent cells carrying MDV-1-ΔU_S_3 BAC DNA to generate MDV-1-MDV-2/U_S_3 BAC. A chimeric MDV-1 virus expressing HVT U_S_3 (MDV-1-HVT/U_S_3) and revertant BAC clones containing MDV-1 U_S_3 (MDV-1-ΔU_S_3_Rev) were generated using the same method (primers 7 to 12 and 13 to 18, respectively; S1 Table). All BAC DNAs were transfected into CEF to produce recombinant viruses.

### Plasmid constructions

All MDV U_S_3, Meq, chHDAC1 and 2 expression plasmids were constructed using pcDNA3.1/Zeo (+) mammalian expression vector (Invitrogen). pGL3-pp14_promoter and pGL3-pp38_promoter plasmids were described previously [25]. All primers used for PCR amplification and gene cloning are listed in S1 Table. The correct sequence of cloned genes was confirmed by sequence analysis.

*U*_*S*_*3 plasmids*. FLAG and HA tagged wild type MDV-1 U_S_3 were amplified from the genome of 686 strain [35] using primers 19, 20, and 21 (S1 Table). The amplified products were digested and cloned into pcDNA. To generate kinase dead MDV-1 U_S_3 (MDV-1_U_S_3-K220A), lysine (K) 220 of MDV-1 U_S_3 was mutated to alanine (A) using primers 22 and 23. Similar overlapping PCR method [36] was used to generate FLAG and HA tagged wild type and kinase dead MDV-2 U_S_3 (S1 Table, primer number 24 to 28) amplified from the genome of SB1 strain (GenBank: HQ840738.1), as well as FLAG and HA tagged wild type and kinase dead HVT U_S_3 (S1 Table, primer number 29 to 33) amplified from the genome of FC-126 strain (GenBank: NC_002641.1).

*Meq plasmids*. HA tagged MDV-1 Meq was amplified from the genome of 686 strain [35] using primers 34 and 35 (S1 Table), followed by digestion and cloning into pcDNA vector.

*chHDAC1 plasmids*. FLAG, HA, and T7 tagged wild type chHDAC1 were amplified from chicken cDNA using primers 36 to 39 (S1 Table), followed by digestion and cloning into pcDNA vector. Single, double, and triple S to A or S to D mutations of chHDAC1 were generated by overlapping PCR using primers 40 to 69 (S1 Table).

*chHDAC2 plasmids*. FLAG, HA, and T7 tagged wild type chHDAC2 were amplified from chicken cDNA using primers 70 to 73 (S1 Table) followed by digestion and cloning into pcDNA vector. Single and double S to A or S to D mutations of chHDAC2 were generated as stated above using primers 74 to 91 (S1 Table).

### Chemicals

Sodium butyrate (NaB) and pimelic diphenylamide 106 (TC-H 106) (Millipore-Sigma) were reconstituted with water or DMSO and used at 1 mM and 10 μM, respectively, to inhibit enzymatic activities of chicken HDACs. Cycloheximide (CHX) (Millipore-Sigma) was reconstituted in DMSO and used at 1 mg/ml to examine the half-life of proteins.

### Immunoprecipitation (IP) and Western blot (WB) analysis

To determine the interaction network of chHDAC1 and 2, as well as the interaction between MDV U_S_3 and chHDAC1 and 2, IP and WB were performed as described previously [9,37]. Briefly, 48 hours after transfection cells were lysed with EBC lysis buffer (50 mM Tris-HCl, 120 mM NaCl, 0.5% NP-40, 50 mM NaF, 200 μM Na_2_VO_4_, 1 mM phenylmethylsulfonyl fluoride, supplemented with protease inhibitors) and 500 μg of whole cell lysates were incubated with mouse anti-FLAG agarose beads overnight at 4°C with gentle rotation. Next day, beads were washed five times and boiled in 2x sodium dodecyl sulfate (SDS) buffer for 5 min. The eluted samples were subjected to SDS-polyacrylamide gel electrophoresis (PAGE) along with 10% cell lysate input (50 μg) and transferred to polyvinylidene fluoride (PVDF) membrane. After blocking with 5% nonfat milk, PVDF membranes were incubated with primary antibody followed by horseradish peroxidase (HRP) conjugated secondary antibody, and visualized with a chemiluminescent substrate.

### Dephosphorylation assay

To confirm that MDV U_S_3 mediates chHDAC1 and 2 phosphorylation, cell lysates were subjected to a dephosphorylation assay [16]. Briefly, 30 μg of whole cell lysates were incubated with or without 20 units of Lambda Protein Phosphatase (Lambda PP, New England Biolabs) in the buffer provided by the manufacturer and supplemented with additional protease inhibitors at 30°C for 15 min. The reaction was stopped by adding 2x SDS buffer, followed by SDS-PAGE and WB as described above.

### Immunofluorescence assay (IFA) and immunohistochemistry (IHC)

IFA was performed to determine the sub-cellular localization of MDV-1 U_S_3, chHDAC1 and 2 in DF-1 cells, and to visualize viral plaques formed by MDV infection in CEF. IHC was performed to determine replication of MDV in the feather follicle epithelium (FFE) of inoculated chickens.

*Transfected DF-1 cells*. IFA of DF-1 cells was carried out as described previously [9]. Briefly, pcDNA-FLAG-MDV-1 U_S_3 plasmid was transfected into DF-1 cells seeded on coverslips using polyethylenimine (PEI, 1 mg/ml) reagent. After 48 hours, cells were fixed and permeabilized prior to blocking in 5% nonfat milk for 1 hour at room temperature. After three washes with phosphate-buffered saline (PBS), cells were incubated with rabbit anti-FLAG antibody and mouse anti-HDAC1 antibody or mouse anti-HDAC2 antibody for 1 hour followed by another hour incubation with goat anti-rabbit-Alexa Fluor 488 antibody and goat anti-mouse-Texas Red antibody, at room temperature. Normal rabbit and mouse IgG were used as negative control to show non-specific staining. 4’,6-diamidino-2-phenylindole (DAPI) was used to stain cell nuclei. Coverslips were mounted on glass slides with ProLong Diamond Antifade Mountant (Thermo Fisher Scientific) and imaged with a Zeiss LSM 780 NLO Multiphoton Microscope.

*Infected CEF*. One day before infection, CEF were seeded in 35 mm cell culture plates. Next day, CEF were treated with NaB (1 mM) or TC-H 106 (10 μM) or mock treated for 6 hours prior to infection with different viruses. After infection, cell culture medium was changed every two days with or without the addition of fresh NaB or TC-H 106. Seven days later, cells were fixed with ice-cold acetone-methanol (3:2) for 5 min, followed by blocking with 5% nonfat milk for 1 hour at room temperature. Cells were probed with MDV pp38 monoclonal antibody or normal mouse IgG followed by goat anti-mouse-Alexa Fluor 488 antibody for 1 hour at room temperature. After three washes, cells were viewed with a fluorescence microscope.

*IHC of feather follicular epithelium (FFE)*. Skin samples from chickens inoculated with recombinant viruses were collected 14 days post inoculation. Tissue samples were fixed in 10% neutral buffered formalin solution for 2 days and transferred to 70% ethanol solution until further use. Formalin fixed and paraffin embedded (FFPE) tissue sections were prepared and mounted on glass slides. Immunostaining was performed using MDV pp38 specific monoclonal antibody and VECTASTAIN ABC kit (Vector Laboratories, Burlingame, CA) as per manufacturer’s protocol. Normal mouse IgG was used as negative control.

### RNA isolation and quantitative reverse transcriptase real time polymerase chain reaction (qRT-PCR)

To determine the role of MDV U_S_3 in regulating the transcription of chHDAC1 and 2 target genes, DF-1 cells were transfected with wild type or kinase dead U_S_3 from MDV-1, MDV-2 and HVT or Ev. Forty-eight hours later, total RNA was isolated using TRIzol reagent (Invitrogen) according to manufacturer’s protocol. 1–5 μg of total RNA was converted to cDNA, followed by qRT-PCR analysis using primers 92 to 101 (S1 Table) and iTaq Universal SYBR Green Supermix (Bio-Rad) in a CFX96 Real time PCR Detection System (Bio-Rad). qRT-PCR results were analyzed using the 2^-ΔΔCT^ method [38].

### Dual luciferase assay

To study the role of chHDAC1 and 2 in regulating the transcriptional activity of the pp14/pp38 bidirectional MDV promoter, 293T cells were co-transfected with plasmids expressing chHDAC1 or 2 or Ev and pGL3-pp14_promoter or pGL3-pp38_promoter. *Renilla* luciferase vector was included as normalization control. Forty-eight hours later, cells were lysed and *Firefly* and *Renilla* luciferase activities were measured according to the manufacturer’s protocol (Promega).

### *In vitro* growth kinetics and plaque area determination

To evaluate the role of MDV U_S_3 in regulating the growth properties and plaque size of MDV, the growth kinetics of parental, chimeric and revertant viruses (MDV-1, MDV-1-ΔU_S_3, MDV-1-ΔU_S_3_Rev, MDV-1- MDV-2/U_S_3, and MDV-1-HVT/U_S_3) were determined as previously described [3]. Briefly, 10^6^ CEF seeded on 60 mm cell culture plates were infected, in duplicate, with parental, chimeric or revertant viruses at 100 plaque-forming units (PFU). On 1, 2, 3, 4, and 5 days post infection, infected CEF were trypsinized, serially diluted 10-fold, and used to infect CEF seeded on 35 mm cell culture plates, in duplicate. Cells were fixed at 7 days post infection and subjected to IFA with mouse anti-pp38 antibody. Plaque numbers were counted for each virus, mean virus titer at each time point calculated, and individual plaque areas measured by Image J software as described previously [5].

### MDV genome copy number

To determine MDV genome replication, qPCR assay was performed to measure MDV genome copy number. Briefly, CEF seeded on 35 mm cell culture plates were infected with 100 PFU of wild type, chimeric or revertant viruses. On 1, 2, 3, 4, and 5 days post infection, cells were harvested for genomic DNA isolation. MDV genome copy number of each virus was determined by qPCR assay with primers specific to MDV *ICP4*, and chicken *GAPDH* as internal control, (primers 102 to 105; S1 Table) and calculated using a standard curve method, as previously described [39]. The relative MDV genome copy number was presented as the ratio of ICP4 to GAPDH copy number.

### Animal experiment 1

To study the role of MDV U_S_3 in regulating MDV replication in chickens, *in vivo* replication properties of chimeric and revertant viruses were determined in spleens of infected chickens. One-day-old chickens were randomly sorted into 6 experimental groups of 15 chickens each. Five groups of chickens were inoculated subcutaneously with 2000 PFU of parental (MDV-1), U_S_3 deletion mutant (MDV-1-ΔU_S_3), U_S_3 revertant (MDV-1-ΔU_S_3_Rev), or U_S_3 chimera (MDV-1- MDV-2/U_S_3, and MDV-1-HVT/U_S_3) viruses; one group remained as uninoculated control. Five chickens were randomly selected from each group at 6, 14, and 28 days post inoculation, and spleens collected for lymphocyte isolation using Histopaque (Sigma) followed by genomic DNA isolation. Viral genome copy number was determined as stated above. At day 14 post inoculation, thymus, bursa, and body weight of 5 chickens per group were measured to evaluate lymphoid organ atrophy. Skin samples from 5 inoculated chickens were collected at 14 days post inoculation for IHC analysis.

### Animal experiment 2

To evaluate the role of MDV U_S_3 on MD pathogenesis, survival rate and tumor development were determined in chickens inoculated with parental, chimeric and revertant viruses. One-day-old chickens were randomly sorted into 6 experimental groups of 12 chickens each. Five groups were inoculated subcutaneously with 2000 PFU of parental (MDV-1), U_S_3 deletion mutant (MDV-1-ΔU_S_3), U_S_3 revertant (MDV-1-ΔU_S_3_Rev), or U_S_3 chimera (MDV-1- MDV-2/U_S_3, and MDV-1-HVT/U_S_3) viruses; one group remained as uninoculated control. Chickens were monitored daily for 10 weeks. All chickens were necropsied at the time of death or at the end of the experiment to evaluate the presence of MDV-specific lesions in visceral organs and nerves.

## Supporting information

S1 Fig MDV U_S_3 mediates the phosphorylation of chHDAC1 and 2 in transfected CEF and DF-1 cells. pcDNA-HA-chHDAC1 (A, C) or pcDNA-HA-chHDAC2 (B, D) were co-transfected with pcDNA FLAG tagged wild type MDV-1, MDV-2, or HVT U_S_3, kinase dead U_S_3 (pcDNA-FLAG-U_S_3-K220A for MDV-1, pcDNA-FLAG-U_S_3-K211A for MDV-2, and pcDNA-FLAG-U_S_3-K212A for HVT), or pcDNA empty vector (Ev) to CEF (A, B) or DF-1 (C, D) cells. Forty-eight hours later, cells were lysed and subjected to Western blot (WB) analysis with FLAG antibody. HSP90 was stained as loading control. (E) CEF cells were transfected with the indicated plasmids for 48 hours, followed by WB with HDAC1, HDAC2, FLAG, and HSP90 antibodies. Protein bands of phosphorylated chHDAC1 (p-chHDAC1) and p-chHDAC2 are marked by arrow.
(PDF)

S2 Fig MDV U_S_3 induces phosphorylation of cellular protein kinase substrates. pcDNA FLAG tagged wild type MDV-1, MDV-2, or HVT U_S_3, pcDNA kinase dead U_S_3 (pcDNA-FLAG-U_S_3-K220A for MDV-1, pcDNA-FLAG-U_S_3-K211A for MDV-2, and pcDNA-FLAG-U_S_3-K212A for HVT), or pcDNA empty vector (Ev) were co-transfected into 293T cells. After 48 hours, whole cell lysates were subjected to Western blot with anti-phospho-PKA substrate antibody (left), anti-phospho-PKC substrate antibody (middle), and anti-phospho-AMPK substrate antibody (right). Staining for FLAG and HSP90 was performed to show U_S_3 expression and protein loading control, respectively.
(PDF)

S3 Fig MDV U_S_3 physically associates with chHDAC1 and 2. pcDNA-FLAG-chHDAC1 and pcDNA empty vector (Ev) (A, C, E), or pcDNA-FLAG-chHDAC2 and pcDNA Ev (B, D, F) were co-transfected with pcDNA HA tagged MDV-1 U_S_3 or MDV-1 U_S_3-K220A (A, B), MDV-2 U_S_3 or MDV-2 U_S_3-K211A (C, D), or HVT U_S_3 or HVT U_S_3-K212A (E, F). Forty-eight hours later, cells were lysed and subjected to immunoprecipitation (IP) with mouse anti-FLAG agarose beads, followed by Western blot (WB) with HA and FLAG antibodies. pcDNA-FLAG-MDV-1-U_S_3, pcDNA-FLAG-MDV-1-U_S_3-K220A, or Ev were co-transfected with pcDNA-HA-chHDAC1 into CEF (G) or DF-1 (H) cells. After 48 hours, cells were lysed and subjected to IP with mouse anti-FLAG agarose beads. WB analysis was performed with HA and FLAG antibodies.
(PDF)

S1 Table List of primers used in MDV-1 BAC mutagenesis and pcDNA plasmid constructions. (PDF)

## References

[1] Davison A. Comments on the phylogenetics and evolution of herpesviruses and other large DNA viruses. Virus Res. 2002;82(1–2):127–32. 10.1016/s0168-1702(01)00400-2 11885939

[2] Tulman ER, Afonso CL, Lu Z, Zsak L, Rock DL, Kutish GF. The genome of a very virulent Marek’s disease virus. J Virol. 2000;74(17):7980–8. 10.1128/jvi.74.17.7980-7988.2000 10933706 PMC112329

[3] Lupiani B, Lee LF, Cui X, Gimeno I, Anderson A, Morgan RW, et al. Marek’s disease virus-encoded Meq gene is involved in transformation of lymphocytes but is dispensable for replication. Proc Natl Acad Sci U S A. 2004;101(32):11815–20. 10.1073/pnas.0404508101 15289599 PMC511057

[4] Trapp S, Parcells MS, Kamil JP, Schumacher D, Tischer BK, Kumar PM, et al. A virus-encoded telomerase RNA promotes malignant T cell lymphomagenesis. J Exp Med. 2006;203(5):1307–17. 10.1084/jem.20052240 16651385 PMC2121211

[5] Schumacher D, Tischer BK, Trapp S, Osterrieder N. The protein encoded by the US3 orthologue of Marek’s disease virus is required for efficient de-envelopment of perinuclear virions and involved in actin stress fiber breakdown. J Virol. 2005;79(7):3987–97. 10.1128/JVI.79.7.3987-3997.2005 15767401 PMC1061555

[6] Schumacher D, McKinney C, Kaufer BB, Osterrieder N. Enzymatically inactive U(S)3 protein kinase of Marek’s disease virus (MDV) is capable of depolymerizing F-actin but results in accumulation of virions in perinuclear invaginations and reduced virus growth. Virology. 2008;375(1):37–47. 10.1016/j.virol.2008.01.026 18304599 PMC2430872

[7] Deruelle MJ, Favoreel HW. Keep it in the subfamily: the conserved alphaherpesvirus US3 protein kinase. J Gen Virol. 2011;92(Pt 1):18–30. 10.1099/vir.0.025593-0 20943887

[8] Kato A, Kawaguchi Y. Us3 Protein Kinase Encoded by HSV: The Precise Function and Mechanism on Viral Life Cycle. Adv Exp Med Biol. 2018;1045:45–62. 10.1007/978-981-10-7230-7_3 29896662

[9] Liao Y, Lupiani B, Bajwa K, Khan OA, Izumiya Y, Reddy SM. Role of Marek’s disease virus encoded US3 serine/threonine protein kinase in regulating MDV Meq and cellular CREB phosphorylation. J Virol. 2020. 10.1128/JVI.00892-20 32581093 PMC7431815

[10] Guise AJ, Budayeva HG, Diner BA, Cristea IM. Histone deacetylases in herpesvirus replication and virus-stimulated host defense. Viruses. 2013;5(7):1607–32. 10.3390/v5071607 23807710 PMC3738950

[11] Segre CV, Chiocca S. Regulating the regulators: the post-translational code of class I HDAC1 and HDAC2. J Biomed Biotechnol. 2011;2011:690848. 10.1155/2011/690848 21197454 PMC3004424

[12] Brunmeir R, Lagger S, Seiser C. Histone deacetylase HDAC1/HDAC2-controlled embryonic development and cell differentiation. Int J Dev Biol. 2009;53(2–3):275–89. 10.1387/ijdb.082649rb 19412887

[13] Pflum MK, Tong JK, Lane WS, Schreiber SL. Histone deacetylase 1 phosphorylation promotes enzymatic activity and complex formation. J Biol Chem. 2001;276(50):47733–41. 10.1074/jbc.M105590200 11602581

[14] Tsai SC, Seto E. Regulation of histone deacetylase 2 by protein kinase CK2. J Biol Chem. 2002;277(35):31826–33. 10.1074/jbc.M204149200 12082111

[15] Khan DH, He S, Yu J, Winter S, Cao W, Seiser C, et al. Protein kinase CK2 regulates the dimerization of histone deacetylase 1 (HDAC1) and HDAC2 during mitosis. J Biol Chem. 2013;288(23):16518–28. 10.1074/jbc.M112.440446 23612983 PMC3675587

[16] Walters MS, Erazo A, Kinchington PR, Silverstein S. Histone deacetylases 1 and 2 are phosphorylated at novel sites during varicella-zoster virus infection. J Virol. 2009;83(22):11502–13. 10.1128/JVI.01318-09 19740981 PMC2772673

[17] Poon AP, Gu H, Roizman B. ICP0 and the US3 protein kinase of herpes simplex virus 1 independently block histone deacetylation to enable gene expression. Proc Natl Acad Sci U S A. 2006;103(26):9993–8. 10.1073/pnas.0604142103 16785443 PMC1502567

[18] Walters MS, Kinchington PR, Banfield BW, Silverstein S. Hyperphosphorylation of histone deacetylase 2 by alphaherpesvirus US3 kinases. J Virol. 2010;84(19):9666–76. 10.1128/JVI.00981-10 20660201 PMC2937806

[19] Shindo K, Kato A, Koyanagi N, Sagara H, Arii J, Kawaguchi Y. Characterization of a Herpes Simplex Virus 1 (HSV-1) Chimera in Which the Us3 Protein Kinase Gene Is Replaced with the HSV-2 Us3 Gene. J Virol. 2016;90(1):457–73. 10.1128/JVI.02376-15 26491159 PMC4702531

[20] Calnek BW. Pathogenesis of Marek’s disease virus infection. Curr Top Microbiol Immunol. 2001;255:25–55. 10.1007/978-3-642-56863-3_2 11217426

[21] McPherson MC, Delany ME. Virus and host genomic, molecular, and cellular interactions during Marek’s disease pathogenesis and oncogenesis. Poult Sci. 2016;95(2):412–29. 10.3382/ps/pev369 26755654 PMC4957504

[22] Benetti L, Roizman B. Herpes simplex virus protein kinase US3 activates and functionally overlaps protein kinase A to block apoptosis. Proc Natl Acad Sci U S A. 2004;101(25):9411–6. 10.1073/pnas.0403160101 15192152 PMC438990

[23] Huang Y, Chen J, Lu C, Han J, Wang G, Song C, et al. HDAC1 and Klf4 interplay critically regulates human myeloid leukemia cell proliferation. Cell Death Dis. 2014;5:e1491. 10.1038/cddis.2014.433 25341045 PMC4237257

[24] Yamaguchi T, Cubizolles F, Zhang Y, Reichert N, Kohler H, Seiser C, et al. Histone deacetylases 1 and 2 act in concert to promote the G1-to-S progression. Genes Dev. 2010;24(5):455–69. 10.1101/gad.552310 20194438 PMC2827841

[25] Levy AM, Izumiya Y, Brunovskis P, Xia L, Parcells MS, Reddy SM, et al. Characterization of the chromosomal binding sites and dimerization partners of the viral oncoprotein Meq in Marek’s disease virus-transformed T cells. J Virol. 2003;77(23):12841–51. 10.1128/jvi.77.23.12841-12851.2003 14610205 PMC262596

[26] Martinez FP, Tang Q. Leucine zipper domain is required for Kaposi sarcoma-associated herpesvirus (KSHV) K-bZIP protein to interact with histone deacetylase and is important for KSHV replication. J Biol Chem. 2012;287(19):15622–34. 10.1074/jbc.M111.315861 22416134 PMC3346108

[27] Karimian A, Ahmadi Y, Yousefi B. Multiple functions of p21 in cell cycle, apoptosis and transcriptional regulation after DNA damage. DNA Repair (Amst). 2016;42:63–71. 10.1016/j.dnarep.2016.04.008 27156098

[28] Hiromura K, Pippin JW, Fero ML, Roberts JM, Shankland SJ. Modulation of apoptosis by the cyclin-dependent kinase inhibitor p27(Kip1). J Clin Invest. 1999;103(5):597–604. 10.1172/JCI5461 10074476 PMC408127

[29] Jerome KR, Fox R, Chen Z, Sears AE, Lee H, Corey L. Herpes simplex virus inhibits apoptosis through the action of two genes, Us5 and Us3. J Virol. 1999;73(11):8950–7. 10.1128/JVI.73.11.8950-8957.1999 10516000 PMC112926

[30] Leopardi R, Van Sant C, Roizman B. The herpes simplex virus 1 protein kinase US3 is required for protection from apoptosis induced by the virus. Proc Natl Acad Sci U S A. 1997;94(15):7891–6. 10.1073/pnas.94.15.7891 9223283 PMC21525

[31] Mounce BC, Mboko WP, Bigley TM, Terhune SS, Tarakanova VL. A conserved gammaherpesvirus protein kinase targets histone deacetylases 1 and 2 to facilitate viral replication in primary macrophages. J Virol. 2013;87(13):7314–25. 10.1128/JVI.02713-12 23616648 PMC3700300

[32] Liao Y, Lupiani B, Bajwa K, Khan OA, Izumiya Y, Reddy SM. Role of Marek’s Disease Virus (MDV)-Encoded US3 Serine/Threonine Protein Kinase in Regulating MDV Meq and Cellular CREB Phosphorylation. J Virol. 2020;94(17). 10.1128/JVI.00892-20 32581093 PMC7431815

[33] Wang ZY, Qin W, Yi F. Targeting histone deacetylases: perspectives for epigenetic-based therapy in cardio-cerebrovascular disease. J Geriatr Cardiol. 2015;12(2):153–64. 10.11909/j.issn.1671-5411.2015.02.010 25870619 PMC4394331

[34] Hernandez R, Brown DT. Growth and maintenance of chick embryo fibroblasts (CEF). Curr Protoc Microbiol. 2010;Appendix 4:4I. 10.1002/9780471729259.mca04is17 20440679

[35] Reddy SM, Sun A, Khan OA, Lee LF, Lupiani B. Cloning of a very virulent plus, 686 strain of Marek’s disease virus as a bacterial artificial chromosome. Avian Dis. 2013;57(2 Suppl):469–73. 10.1637/10444-110412-ResNote.1 23901763

[36] Ho SN, Hunt HD, Horton RM, Pullen JK, Pease LR. Site-directed mutagenesis by overlap extension using the polymerase chain reaction. Gene. 1989;77(1):51–9. 10.1016/0378-1119(89)90358-2 2744487

[37] Sambrook J, Russell DW. Identification of associated proteins by coimmunoprecipitation. CSH Protoc. 2006;2006(1). 10.1101/pdb.prot3898 22485357

[38] Nakayama K. cAMP-response element-binding protein (CREB) and NF-kappaB transcription factors are activated during prolonged hypoxia and cooperatively regulate the induction of matrix metalloproteinase MMP1. J Biol Chem. 2013;288(31):22584–95. 10.1074/jbc.M112.421636 23775082 PMC3829345

[39] Heidari M, Delekta PC. Transcriptomic Analysis of Host Immune Response in the Skin of Chickens Infected with Marek’s Disease Virus. Viral Immunol. 2017;30(5):377–87. 10.1089/vim.2016.0172 28410454

