# Supplementary material for: Marek’s disease virus US3 protein kinase phosphorylates chicken HDAC 1 and 2 and regulates viral replication and pathogenesis
Source: PLoS Pathog. 2021 Feb 17;17(2):e1009307. doi: 10.1371/journal.ppat.1009307 (PMC7920345; doi:10.1371/journal.ppat.1009307)
Supplement: S1 Fig — pcDNA-HA-chHDAC1 (A, C) or pcDNA-HA-chHDAC2 (B, D) were co-transfected with pcDNA FLAG tagged wild type MDV-1, MDV-2, or HVT US3, kinase dead US3 (pcDNA-FLAG-US3-K220A for MDV-1, pcDNA-FLAG-US3-K211A for MDV-2, and pcDNA-FLAG-US3-K212A for HVT), or pcDNA empty vector (Ev) to CEF (A, B) or DF-1 (C, D) cells. Forty-eight hours later, cells were lysed and subjected to Western blot (WB) analysis with FLAG antibody. HSP90 was stained as loading control. (E) CEF cells were transfected with the indicated plasmids for 48 hours, followed by WB with HDAC1, HDAC2, FLAG, and HSP90 antibodies. Protein bands of phosphorylated chHDAC1 (p-chHDAC1) and p-chHDAC2 are marked by arrow. (PDF) [file ppat.1009307.s001.pdf]

# Supplementary Figures

**S1 Fig**

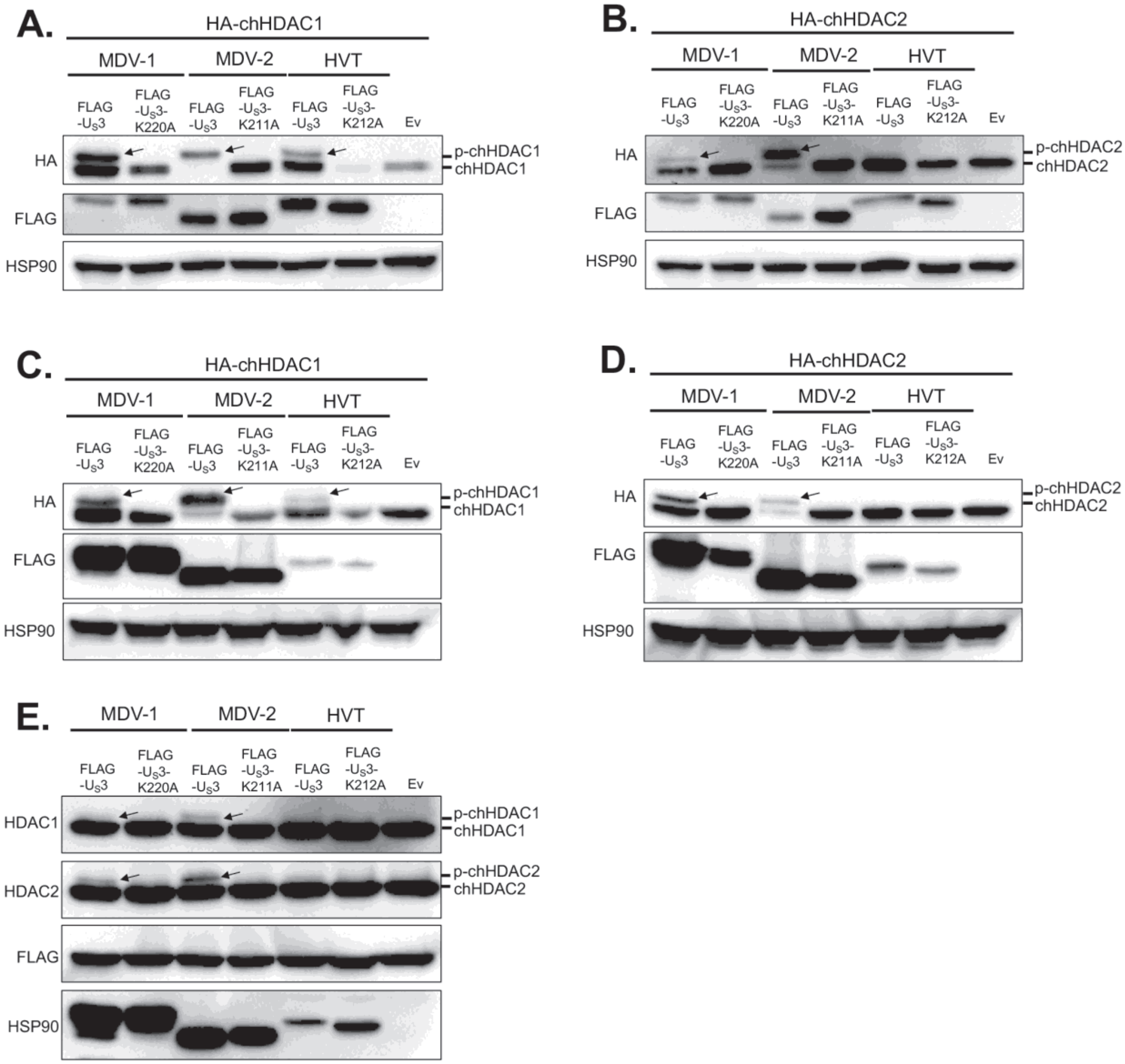


**S1 Fig. MDV $U_S3$ mediates the phosphorylation of chHDAC1 and 2 in transfected CEF and DF-1 cells.** pcDNA-HA-chHDAC1 (A, C) or pcDNA-HA-chHDAC2 (B, D) were co-transfected with pcDNA FLAG tagged wild type MDV-1, MDV-2, or HVT $U_S3$, kinase dead $U_S3$ (pcDNA-FLAG-$U_S3$-K220A for MDV-1, pcDNA-FLAG-$U_S3$-K211A for MDV-2, and pcDNA-FLAG-$U_S3$-K212A for HVT), or pcDNA empty vector (Ev) to CEF (A, B) or DF-1 (C, D) cells. Forty-eight hours later, cells were lysed and subjected to Western blot (WB) analysis with FLAG antibody. HSP90 was stained as loading control. (E) CEF cells were transfected with the indicated plasmids for 48 hours, followed by WB with HDAC1, HDAC2, FLAG, and HSP90 antibodies. Protein bands of phosphorylated chHDAC1 (p-chHDAC1) and p-chHDAC2 are marked by arrow.
