# Supplementary material for: Marek’s disease virus US3 protein kinase phosphorylates chicken HDAC 1 and 2 and regulates viral replication and pathogenesis
Source: PLoS Pathog. 2021 Feb 17;17(2):e1009307. doi: 10.1371/journal.ppat.1009307 (PMC7920345; doi:10.1371/journal.ppat.1009307)
Supplement: S2 Fig — pcDNA FLAG tagged wild type MDV-1, MDV-2, or HVT US3, pcDNA kinase dead US3 (pcDNA-FLAG-US3-K220A for MDV-1, pcDNA-FLAG-US3-K211A for MDV-2, and pcDNA-FLAG-US3-K212A for HVT), or pcDNA empty vector (Ev) were co-transfected into 293T cells. After 48 hours, whole cell lysates were subjected to Western blot with anti-phospho-PKA substrate antibody (left), anti-phospho-PKC substrate antibody (middle), and anti-phospho-AMPK substrate antibody (right). Staining for FLAG and HSP90 was performed to show US3 expression and protein loading control, respectively. (PDF) [file ppat.1009307.s002.pdf]

S2 Fig

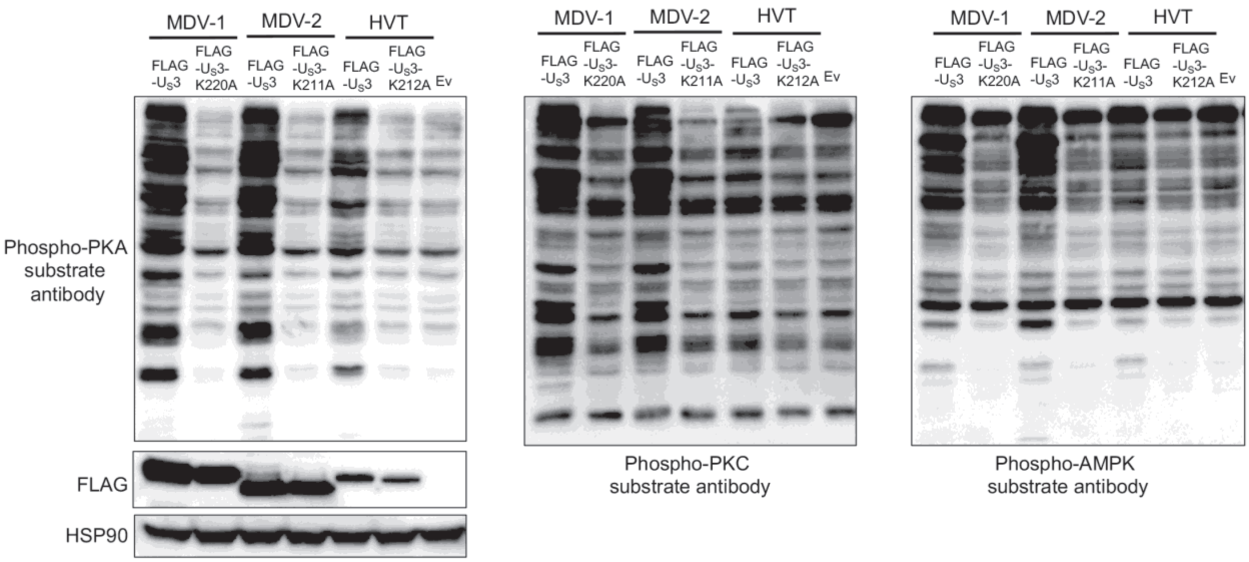

S2 Fig. MDV U_S3 induces phosphorylation of cellular protein kinase substrates. pcDNA FLAG tagged wild type MDV-1, MDV-2, or HVT U_S3, pcDNA kinase dead U_S3 (pcDNA-FLAG-U_S3-K220A for MDV-1, pcDNA-FLAG-U_S3-K211A for MDV-2, and pcDNA-FLAG-U_S3-K212A for HVT), or pcDNA empty vector (Ev) were co-transfected into 293T cells. After 48 hours, whole cell lysates were subjected to Western blot with anti-phospho-PKA substrate antibody (left), anti-phospho-PKC substrate antibody (middle), and anti-phospho-AMPK substrate antibody (right). Staining for FLAG and HSP90 was performed to show U_S3 expression and protein loading control, respectively.
