# Supplementary material for: Marek’s disease virus US3 protein kinase phosphorylates chicken HDAC 1 and 2 and regulates viral replication and pathogenesis
Source: PLoS Pathog. 2021 Feb 17;17(2):e1009307. doi: 10.1371/journal.ppat.1009307 (PMC7920345; doi:10.1371/journal.ppat.1009307)
Supplement: S3 Fig — pcDNA-FLAG-chHDAC1 and pcDNA empty vector (Ev) (A, C, E), or pcDNA-FLAG-chHDAC2 and pcDNA Ev (B, D, F) were co-transfected with pcDNA HA tagged MDV-1 US3 or MDV-1 US3-K220A (A, B), MDV-2 US3 or MDV-2 US3-K211A (C, D), or HVT US3 or HVT US3-K212A (E, F). Forty-eight hours later, cells were lysed and subjected to immunoprecipitation (IP) with mouse anti-FLAG agarose beads, followed by Western blot (WB) with HA and FLAG antibodies. pcDNA-FLAG-MDV-1-US3, pcDNA-FLAG-MDV-1-US3-K220A, or Ev were co-transfected with pcDNA-HA-chHDAC1 into CEF (G) or DF-1 (H) cells. After 48 hours, cells were lysed and subjected to IP with mouse anti-FLAG agarose beads. WB analysis was performed with HA and FLAG antibodies. (PDF) [file ppat.1009307.s003.pdf]

S3 Fig

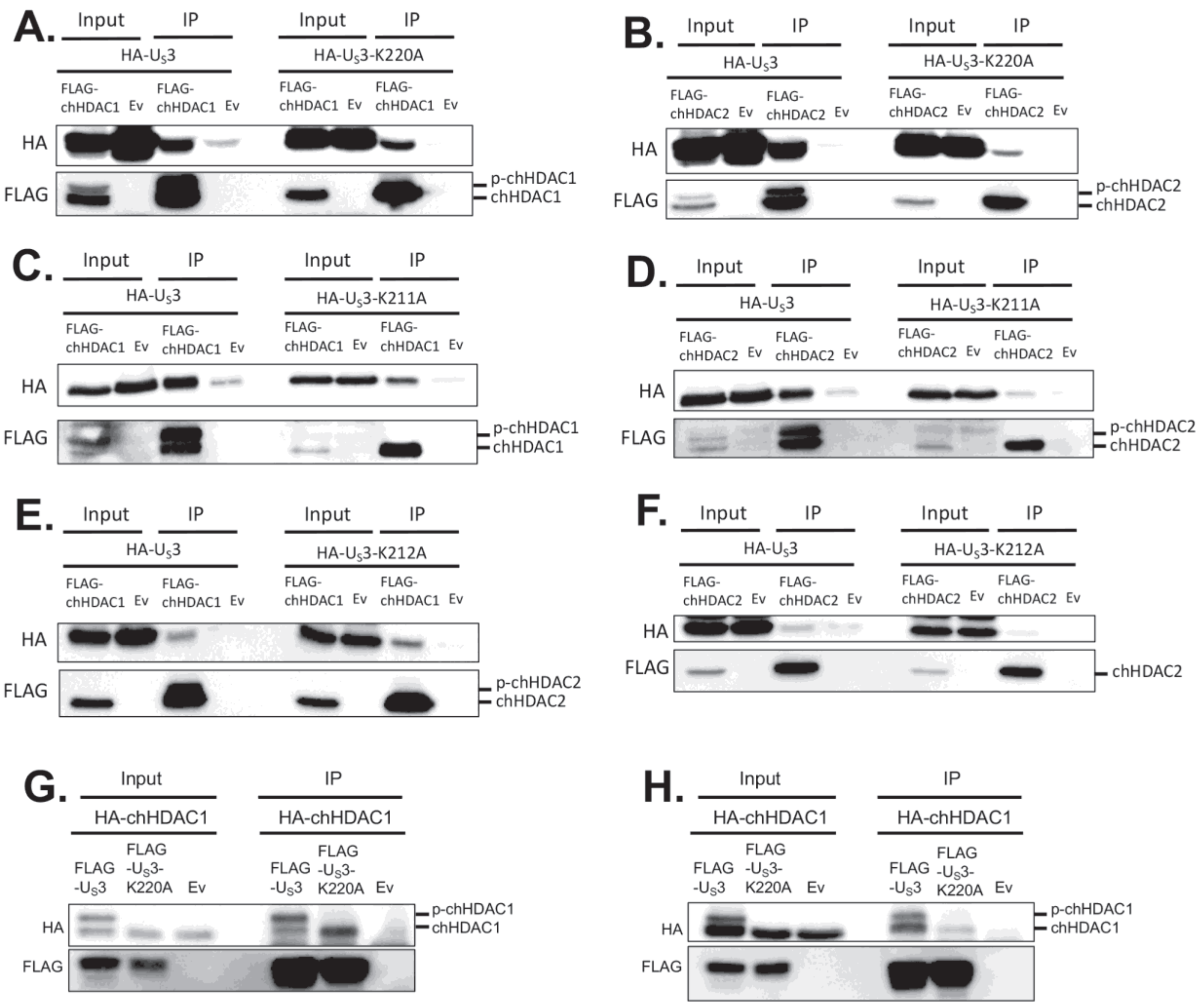

S3 Fig. MDV U_S3 physically associates with chHDAC1 and 2. pcDNA-FLAG-chHDAC1 and pcDNA empty vector (Ev) (A, C, E), or pcDNA-FLAG-chHDAC2 and pcDNA Ev (B, D, F) were co-transfected with pcDNA HA tagged MDV-1 U_S3 or MDV-1 U_S3-K220A (A, B), MDV-2 U_S3 or MDV-2 U_S3-K211A (C, D), or HVT U_S3 or HVT U_S3-K212A (E, F). Forty-eight hours later, cells were lysed and subjected to immunoprecipitation (IP) with mouse anti-FLAG agarose beads, followed by Western blot (WB) with HA and FLAG antibodies. pcDNA-FLAG-MDV-1-U_S3, pcDNA-FLAG-MDV-1-U_S3-K220A, or Ev were co-transfected with pcDNA-HA-chHDAC1 into CEF (G) or DF-1 (H) cells. After 48 hours, cells were lysed and subjected to IP with mouse anti-FLAG agarose beads. WB analysis was performed with HA and FLAG antibodies.
