# Supplementary material for: Marek’s disease virus US3 protein kinase phosphorylates chicken HDAC 1 and 2 and regulates viral replication and pathogenesis
Source: PLoS Pathog. 2021 Feb 17;17(2):e1009307. doi: 10.1371/journal.ppat.1009307 (PMC7920345; doi:10.1371/journal.ppat.1009307)
Supplement: S1 Table — (PDF) [file ppat.1009307.s004.pdf]

S1 Table. List of primers used in MDV-1 BAC mutagenesis and pcDNA plasmid constructions.

Primer number	Primer name	Sequence (5' to 3')	Purpose
1	MDV-2/U_S3_F	GATC**GAATTC**ATGGAAACTAACGAGCTTTC	Generation of MDV-1-MDV-2/US3 BAC
2	MDV-2/U_S3_R	GATC**GGATCC**TTAGGGAACAAATAGGGCCTGGA	
3	MDV-2/U_S3Kan-F	GATC**GATATC**TGGAGTGCCGGGCTTGTGTTATTCGAGATGTCTGCCAAAAAAAGGACACTAGGATGACGACGATAAGTAGGG	
4	MDV-2/U_S3Kan-R	GATC**GATATC**CAACCAATTAACCAATTCTGATTAG	
5	MDV-2/U_S3-T-F	TTATACTCTGGTAGAATATGAAACAGGGTTAAAACTAGGTAATAGACTGGATGGAAACTAACGAGCTTTC	
6	MDV-2/U_S3-T-R	GCGTAGTATATATTATAAAATGAATCATTGAAGTTATTTTTGACGGGTGTTTAGGGAACAAATAGGGCCTGGA	
7	HVT/U_S3_F	GATC**GAATTC**ATGGAAGTAGATGTTGAGTC	Generation of MDV-1-HVT/US3 BAC
8	HVT/U_S3_R	GATC**GGATCC**TTAGACACTGTCAGAGGGTGTGATA	
9	HVT/U_S3Kan-F	GATC**GATATC**AGGGAAGTGGATATTTTAAAAACCATCTCACATAAATCAATTATAAAATTAGGATGACGACGATAAGTAGGG	
10	HVT/U_S3Kan-R	GATC**GATATC**CAACCAATTAACCAATTCTGATTAG	
11	HVT/U_S3-T-F	TTATACTCTGGTAGAATATGAAACAGGGTTAAAACTAGGTAATAGACTGGATGGAAGTAGATGTTGAGTC	
12	HVT/U_S3-T-R	GCGTAGTATATATTATAAAATGAATCATTGAAGTTATTTTTGACGGGTGTTTAGACACTGTCAGAGGGTGTGATA	
13	MDV-1/U_S3_F	GATC**GAATTC**ATGTCTTCGAGTCCGGAGGC	Generation of MDV-1-ΔUS3_Rev BAC
14	MDV-1/U_S3_R	GATC**GGATCC**TTACATATGAGCGGCAGTTA	
15	MDV-1/U_S3Kan-F	GATC**GATATC**ATGGGACCATTGCCACTAAATCAAATAATTACGATAGAACGGGGTTTGCTAGGATGACGACGATAAGTAGGG	
16	MDV-1/U_S3Kan-R	GATC**GATATC**CAACCAATTAACCAATTCTGATTAG	
17	MDV-1/U_S3-T-F	TTATACTCTGGTAGAATATGAAACAGGGTTAAAACTAGGTAATAGACTGGATGTCTTCGAGTCCGGAGGC	
18	MDV-1/U_S3-T-R	GCGTAGTATATATTATAAAATGAATCATTGAAGTTATTTTTGACGGGTGTTTACATATGAGCGGCAGTTATCG	
19	MDV-1_U_S3-FLAG-F	TC**GGATCC**ATG***GACTACAAAGACGATGACGACAAG***TCTTCGAGTCCGGAGGCA	FLAG and HA tagged wild type and kinase dead MDV-1 US3 plasmids
20	MDV-1_U_S3-HA-F	TC**GGATCC**ATG***TACCCATACGATGTTCCAGATTACGCT***TCTTCGAGTCCGGAGGCA	
21	MDV-1_U_S3-R	TC**GAATTC**TTACATATGAGCGGCAGTTA	
22	MDV-1_U_S3-K220A-F	TGATGTAGCAACTGAAAATA	
23	MDV-1_U_S3-K220A-R	TATTTTCAGTTGCTACATCA	
24	MDV-2_U_S3-FLAG-F	GATC**GGATCC**ATG***GACTACAAAGACGATGACGACAAG***gaaactaacgagctttct	FLAG and HA tagged wild type and kinase dead MDV-2 US3 plasmids
25	MDV-2_U_S3-HA-F	GATC**GGATCC**ATG***TACCCATACGATGTTCCAGATTACGCT***gaaactaacgagctttct	
26	MDV-2_U_S3-R	GATC**GAATTC**tcagggaacaaatagggcctggagatt	
27	MDV-2_U_S3-K211A-F	cgcgatgtaGCgacagaaaac	
28	MDV-2_U_S3-K211A-R	gttttctgtcGCtacatcgcg	
29	HVT_U_S3-FLAG-F	GATC**GGATCC**ATG***GACTACAAAGACGATGACGACAAG***gaagtagatgttgagtct	FLAG and HA tagged wild type and kinase dead HVT US3 plasmids
30	HVT_U_S3-HA-F	GATC**GGATCC**ATG***TACCCATACGATGTTCCAGATTACGCT***gaagtagatgttgagtct	
31	HVT_U_S3-R	GATC**GAATTC**ttagacactgtcagagggtgt	
32	HVT_U_S3-K212A-F	cgagacgtaGCgacggagaat	
33	HVT_U_S3-K212A-R	attctccgtcGCtacgtctcg	
34	MDV-1_Meq-HA-F	TC**GCTAGC**ATG***TACCCATACGATGTTCCAGATTACGCT***TCTCAGGAGCCAGAGCCG	HA tagged MDV-1 Meq plasmid
35	MDV-1_Meq-R	GATC**GCGGCCGC**TCAGGGTCTCCCGTCACCTG	
36	chHDAC1_FLAG-F	gatc**GCTAGC**atg***GACTACAAAGACGATGACGACAAG***gcgctgacgcaggggac	FLAG, HA, and T7 tagged wild type chHDAC1 plasmids
37	chHDAC1_HA-F	gatc**GCTAGC**atg***TACCCATACGATGTTCCAGATTACGCT***gcgctgacgcaggggac	
38	chHDAC1_T7-F	tc**GCTAGC**atg***ATGGCTAGCATGACTGGTGGACAGCAAATGGGT***gcgctgacgcaggggga	
39	chHDAC1-R	gatc**GCGGCCGC**ttaggtggattttgtctctt	
40	chHDAC1-S393A-F	gaagacGCGggagatgaaga	Single, double, and triple mutations of chHDAC1 plasmids
41	chHDAC1-S393A-R	tcttcatctccCGCgtcttc	
42	chHDAC1-S406A-F	gcattGCGatccgcaattct	
43	chHDAC1-S406A-R	agaattgcggatCGCaatgc	
44	chHDAC1-S410A-F	ccgcaatGCGgataagagaat	
45	chHDAC1-S410A-R	attctcttatcCGCattgcgg	
46	chHDAC1-S415A-F	gagaataGCGtgtgatgaag	
47	chHDAC1-S415A-R	cttcatcacaCGCtattctc	
48	chHDAC1-S421A-F	tgaagaattcGCGgactctg	
49	chHDAC1-S421A-R	cagagtcCGCgaattcttca	
50	chHDAC1-S423A-F	tctgacGCGgaagatgaagg	
51	chHDAC1-S423A-R	ccttcatcttcCGCgtcaga	
52	chHDAC1-T445A-R	gatc**GCGGCCGC**ttaggtggattttgtctcttccttcacccccttgggttcggctttctcctcttttgctttttcctcttctttctcatccttcttctcctcctcctcctttttcctcctcCGCtttcacacgcttggccttc	
53	chHDAC1-T477A-R	gatc**GCGGCCGC**ttaGGTGGAtttCGCctcttccttca	
54	chHDAC1-S479A-R	gatc**GCGGCCGC**ttaGGTCGCtttTGTctcttccttca	
55	chHDAC1-T480A-R	gatc**GCGGCCGC**ttaCGCGGAtttTGTctcttccttca	
56	chHDAC1-S406/410A-F	cctgagaagcgcattGCGatccgcaatGCGgataagagaatatcctgtgatgaagaattc	
57	chHDAC1-S406/410A-R	gaattcttcatcacaggatattctcttatcCGCattgcggatCGCaatgcgcttctcagg	
58	chHDAC1-S406/410/415A-F	cctgagaagcgcattGCGatccgcaatGCGgataagagaataGCGtgtgatgaagaattc	
59	chHDAC1-S406/410/415A-R	gaattcttcatcacaCGCtattctcttatcCGCattgcggatCGCaatgcgcttctcagg	
60	chHDAC1-S406D-F	cctgagaagcgcattGACatccgcaattct	
61	chHDAC1-S406D-R	agaattgcggatGTCaatgcgcttctcagg	
62	chHDAC1-S410D-F	catttcaatccgcaatGACgataagagaat	
63	chHDAC1-S410D-R	attctcttatcGTCattgcggattgaaatg	
64	chHDAC1-S415D-F	gagaataGACtgtgatgaag	
65	chHDAC1-S415D-R	cttcatcacaGTCtattctc	
66	chHDAC1-S406/410D-F	cctgagaagcgcattGACatccgcaatGACgataagagaatatcctgtgatgaagaattc	

	A	B	C	D
70	67	chHDAC1-S406/410D-R	gaattcttcatcacaggatattctcttatcGTCattgcggatGTCaatgcgcttctcagg	
71	68	chHDAC1-S406/410/415D-F	cctgagaagcgcattGACatccgcaatGACgataagagaataGACtgtgatgaagaattc	
72	69	chHDAC1-S406/410/415D-R	gaattcttcatcacaGTCtattctcttatcGTCattgcggatGTCaatgcgcttctcagg	
73	70	chHDAC2_FLAG-F	gatc**GCTAGC**atg***GACTACAAAGACGATGACGACAAG***gcgtacagtcagggcgg	
74	71	chHDAC2_HA-F	gatc**GCTAGC**atg***TACCCATACGATGTTCCAGATTACGCT***gcgtacagtcagggcgg	FLAG, HA, and T7 tagged wild type chHDAC2 plasmids
75	72	chHDAC2_T7-F	tc**GCTAGC**atg***ATGGCTAGCATGACTGGTGGACAGCAAATGGGT***gcgtacagtcagggcg	
76	73	chHDAC2-R	gatc**GCGGCCGC**tcaaggattgctgagctgtt	
77	74	chHDAC2-S394A-F	catgaagatGCGggagatga	
78	75	chHDAC2-S394A-R	tcatctccCGCatcttcatg	
79	76	chHDAC2-S407A-F	aacgcattGCGattcgagca	
80	77	chHDAC2-S407A-R	tgctcgaatCGCaatgcgtt	
81	78	chHDAC2-S411A-F	tattcgagcaGCGgataagc	
82	79	chHDAC2-S411A-R	gcttatcCGCtgctcgaata	
83	80	chHDAC2-S422A-F	tgatgaggagtttGCGgact	
84	81	chHDAC2-S422A-R	agtcCGCaaactcctcatca	
85	82	chHDAC2-S424A-F	ttttcagacGCGgaagatga	Single and double mutations of chHDAC2 plasmids
86	83	chHDAC2-S424A-R	tcatcttcCGCgtctgaaaa	
87	84	chHDAC2-S407/411A-F	gatccagacaaacgcattGCGattcgagcaGCGgataagcgcattgcctg	
88	85	chHDAC2-S407/411A-R	caggcaatgcgcttatcCGCtgctcgaatCGCaatgcgtttgtctggatc	
89	86	chHDAC2-S407D-F	aacgcattGACattcgagca	
90	87	chHDAC2-S407D-R	tgctcgaatGTCaatgcgtt	
91	88	chHDAC2-S411D-F	tattcgagcaGACgataagc	
92	89	chHDAC2-S411D-R	gcttatcGTCtgctcgaata	
93	90	chHDAC2-S407/411D-F	gatccagacaaacgcattGACattcgagcaGACgataagcgcattgcctg	
94	91	chHDAC2-S407/411D-R	caggcaatgcgcttatcGTCtgctcgaatGTCaatgcgtttgtctggatc	
95	92	chHDAC1_qPCR_F	GGATGAAGAAGAAGAAGATCC	
96	93	chHDAC1_qPCR_R	GATAACTATGCACTGACAGG	
97	94	chHDAC2_qPCR_F	AAGGTGGACGGCGAAATG	
98	95	chHDAC2_qPCR_R	GATACGGTCCATGCCAAATAG	
99	96	chp21_qPCR_F	CGTAGACCACGAGCAGATCC	Gene expression analysis
100	97	chp21_qPCR_R	AGGACCCTCTCCCACTTGAA	
101	98	chp27_qPCR_F	AAACGGGAATTGCCAAACCG	
102	99	chp27_qPCR_R	TGACTGCCAGCAACATCAGT	
103	100	chGAPDH_qPCR_F	GTCAACGGATTTGGCCGTAT	
104	101	chGAPDH_qPCR_R	CCACTTGGACTTTGCCAGAGA	
105	102	ICP4_F	CGCCACACGAGAACACAATG	
106	103	ICP4_R	GGTTGGAGTAGAGCTGCAACTGT	MDV genome copy number
107	104	chGAPDH_F	GTCAACGGATTTGGCCGTAT	
108	105	chGAPDH_R	CCACTTGGACTTTGCCAGAGA	
109				
110	* The sequences that highlighted in bold are restriction enzyme sites.			
111	** The sequences that underlined were used to amplify the *Kan* gene cassette from pEPKan-S plasmid.			
112	*** The sequences that highlighted in bold italics are the tag sequences.			
